# Non-insulin-based insulin resistance indexes in predicting atrial fibrillation recurrence following ablation: a retrospective study

**DOI:** 10.1186/s12933-024-02158-6

**Published:** 2024-02-28

**Authors:** Zhe Wang, Haoming He, Yingying Xie, Jiaju Li, Fangyuan Luo, Zhaowei Sun, Shuwen Zheng, Furong Yang, Xuexi Li, Xiaojie Chen, Yingwei Chen, Yihong Sun

**Affiliations:** 1 Department of Cardiology, China-Japan Friendship Hospital (Institute of Clinical Medical Sciences), Chinese Academy of Medical Sciences & Peking Union Medical College, No.2 East Yinghua Road, Chaoyang District, Beijing, 100029 China; 2 Department of Cardiology, The First Affiliated Hospital of Zhengzhou University, Zhengzhou, China; 3 Department of Cardiology, Beijing University of Chinese Medicine China-Japan Friendship School of Clinical Medicine, Beijing, China

**Keywords:** Atrial fibrillation, Triglyceride glucose-body mass index, Metabolic score for insulin resistance, Radiofrequency ablation, Recurrence

## Abstract

**Background:**

Insulin resistance (IR) is involved in the pathophysiological processes of arrhythmias. Increasing evidence suggests triglyceride and glucose (TyG) index, metabolic score for insulin resistance (METS-IR), triglyceride glucose-body mass index (TyG-BMI), and triglyceride to high-density lipoprotein cholesterol (TG/HDL-C) ratio are simple and reliable surrogates for IR. Although they have been associated with atrial fibrillation (AF), evidence supporting this is limited. Here, this is the first study to investigate the association between TyG-BMI index and AF recurrence following radiofrequency catheter ablation (RFCA). The performance of the four non-insulin-based IR indexes in predicting AF recurrence after ablation was explored.

**Methods:**

A total of 2242 AF patients who underwent a de novo RFCA between June 2018 to January 2022 at two hospitals in China were included in this retrospective study. The predictive values of IR indexes for AF recurrence after ablation were assessed.

**Results:**

During 1-year follow-up, 31.7% of patients experienced AF recurrence. The multivariable analysis revealed that TyG index, METS-IR, and TyG-BMI index were independent risk factors for AF recurrence. Restricted cubic spline analysis revealed a connection between METS-IR, TyG-BMI index, and AF recurrence (*P* < 0.001). Furthermore, incorporating the METS-IR or TyG-BMI index to the basic risk model with fully adjusted factors considerably enhanced the forecast of AF recurrence, as demonstrated by the C-statistic, continuous net reclassification improvement, and integrated discrimination improvement.

**Conclusions:**

TyG index, METS-IR, and TyG-BMI index were independently associated with AF recurrence following ablation. Among the four non-insulin-based IR indexes, TyG-BMI had the highest predictive value, followed by METS-IR.

**Supplementary Information:**

The online version contains supplementary material available at 10.1186/s12933-024-02158-6.

## Introduction

Atrial fibrillation (AF) is one of the common cardiovascular diseases in clinical practice, which can lead to heart failure, stroke, and other complications. The pathogenesis of AF is very complex, and radiofrequency catheter ablation (RFCA) is the main treatment method, but there is a certain recurrence rate after ablation [1]. The recurrence rates of paroxysmal AF (PAF) and persistent AF (PeAF) were 10-30% and 25–35%, respectively, during 1-year follow-up after ablation [2]. Therefore, early and optimized risk stratification is essential to improve the prognosis of AF following ablation.

Insulin resistance (IR) is a pathological condition in which the body’s tissues or cells have a diminished response to insulin as a potential mechanism for abnormal glucose metabolism, and some populations have a susceptibility to impaired glucose tolerance even if they are not obese and may develop IR [3–5]. Although hyperinsulinemic-euglycemic clamp is considered the gold-standard method for evaluating IR, its clinical application is limited by cost and complexity [6]. Interestingly, convenient and validated measures for assessing insulin resistance have been established, such as the metabolic score for triglyceride-glucose (TyG) index, metabolic score for insulin resistance (METS-IR), triglyceride glucose-body mass index (TyG-BMI), and triglyceride to high-density lipoprotein cholesterol (TG/HDL-C) ratio [7]. Moreover, increasing research has revealed indicators of IR to be valuable predictors for the severity and prognosis of diabetes mellitus (DM)、heart failure、coronary heart disease (CHD) [8–10].

At present, there is limited research in investigating the relationship between these IR indexes and AF recurrence following ablation [11]. The predictive value of TyG-BMI index for recurrence after RFCA in patients with AF has not been studied. This study first aimed to investigate the relationship between AF recurrence after RFCA and TyG-BMI index. Furthermore, we sought to compare the values of TyG index, METS-IR, TyG-BMI index, and TG/HDL-C ratio in predicting AF recurrence following ablation.

## Methods

### Study design and populations

We conducted a retrospective observational study from June 2018 to January 2022 at teaching Hospitals of China-Japan Friendship Hospital and First Affiliated Hospital of Zhengzhou University. All enrolled AF patients were admitted to hospital for the first RFCA treatment. The exclusion criteria were as follows: (1) hypertrophic cardiomyopathy; (2) advanced valvular heart disease; (3) end-stage renal disease; (4) thyroid dysfunction. We further excluded patients who were suspected of having familial hypertriglyceridemia (TG ≥ 5.65 mmol/L) and lacking necessary data for IR indexes calculation. Patients who died or lost to follow-up were also excluded. The research followed the guidelines of the Strengthening the Reporting of Observational Studies in Epidemiology (STROBE) statement. The study protocol adhered to the principles of the Declaration of Helsinki and obtained approval by the local ethical review board (2023-KY-0327). In this study, all enrolled patients were exempted from informed consent.

### Data collection and definitions

Data were collected from the electronic medical records of the two participating centers by certified study coordinators. We designed standardized spreadsheets to collect these retrospective data. The initial demographic and clinical data included age, sex, current smoking, current drinking, DM, hypertension, hyperlipidaemia, CHD, history of stroke/transient ischemic attack (TIA), heart failure, duration of AF, type of AF (PAF or PeAF).

Blood samples were collected from the patient’s peripheral veins after fasting for more than 8 h before ablation during hospitalization. Laboratory tests were performed to obtain measurements including white blood cells, platelet (PLT), uric acid (UA), creatinine (Cr), fasting blood glucose (FBG), glycated haemoglobin (HbA1c), total cholesterol (TC), triglycerides (TG), low-density lipoprotein cholesterol (LDL-C), high-density lipoprotein cholesterol (HDL-C). The information regarding RFCA comprised the percentage of linear ablation as well as superior vena cava (SVC) isolation. In addition, documentation was made regarding echocardiographic assessment of left atrial (LA) diameter, left ventricular end-diastolic diameter (LVEDD), and left ventricular ejection fraction (LVEF), and usage of medications before abaltion, which included antiarrhythmic drugs (AADs) therapy, angiotensin-converting enzyme inhibitor (ACEI) or angiotensin receptor blocker (ARB), and statin. Data required for calculating the CHA_2_DS_2_-VASc score was derived from hospital admission records [12].

Hypertension was defined as meeting any of the following criteria: systolic blood pressure ≥ 140 mmHg, diastolic blood pressure ≥ 90 mmHg, or use of anti-hypertensive medication. DM was defined as the use of oral hypoglycaemic drugs or insulin, or HbA1c level ≥ 6.5% on admission. CHD was defined as luminal stenosis of ≥ 50% in at least one major coronary artery. PAF was AF lasting less than 7 days, usually less than 48 h [13]. PeAF was AF lasting longer than 7 days [14]. The duration of AF was calculated by the time from the date of initial symptom onset or first diagnosis of AF to the RFCA date. Duration of atrial fibrillation as a binary variable based on its median value (24 months). Body mass index (BMI) was calculated as weight (kg) divided by the square of height (m^2^). The IR indexes were calculated as the following formula: TyG index = Ln [TG (mg/dL)×FBG (mg/dL) ÷ 2]; TyG-BMI index = TyG×BMI (kg/m^2^); TG/HDL-C = TG (mg/dl) ÷ HDL-C (mg/dl); METS-IR = Ln [(2×FBG (mg/dl)) + TG (mg/ dl)]×BMI (kg/m^2^) ÷ Ln [HDL-C (mg/dl)] [15].

### Ablation protocol

The RFCA procedure has been described before [16, 17]. Briefly, under the guidance of the CARTO system (Johnson & Johnson Medical, Biosense Webster, Inc., Irvine, CA, USA), circumferential pulmonary vein isolation (CPVI) was performed in all AF patients. As an extra-PV ablation, additional procedures such as linear ablation (roof line, tricuspid isthmus linear, and mitral valve isthmus linear), and complex fractionated atrial electrograms ablation were performed, especially in patients with PeAF. Isolation of superior vena cava (SVC) was performed if induced tachycardia from SVC or the potential of SVC was active. At the end of the ablation, if re-connection of pulmonary vein was observed, a pulmonary vein isolation was performed.

### Outcomes and follow‑up

The primary outcome was the recurrence of AF after ablation during 1 year follow-up. The definition of AF recurrence was any atrial tachyarrhythmia lasting for more than 30 s in electrocardiogram or Holter monitoring after the 3-month blanking period. All patients were prescribed AADs for 3 months after ablation to prevent early recurrence. The use of AADs was subsequently determined by physicians and patients. Patients were scheduled for follow-up at three-month intervals within the first year after ablation in the outpatient setting and telephone. Each follow-up visit included 12-lead electrocardiogram, 24-hour Holter monitoring, and clinical assessment. Patients who had any symptoms related to AF were required to complete an additional outpatient visit.

### Statistical analysis

Continuous variables were described as the mean ± standard deviation or the median with interquartile range depending on the normality of the data distribution, and comparisons between groups were performed the Student’s t-test. Categorical variables were presented as frequencies (percentages), and comparisons between groups were carried out using the chi-square (χ^2^) test. A receiver operating characteristic (ROC) curve was used for diagnostic value analysis in four IR indexes. Kaplan-Meier method was used to create cumulative curves for recurrence of AF following ablation, and the log-rank test was utilized to differentiate between the curves of the groups. Multivariable Cox proportional hazard models were constructed to assess the correlation between IR indexes and AF recurrence. The first model remained unchanged, whereas second model included age and gender as factors. The third model was completely calibrated. Table 1 contained the candidate variables, with the third model including confounders that were statistically significant. However, variables in the score formula for IR indexes were not together in the multivariate Cox analysis model. The hazard ratio (HR) along with a corresponding 95% confidence interval (CI) were presented as the outcomes obtained from the Cox regression model. Moreover, an analysis using restricted cubic spline (RCS) was performed with four knots to identify any possible nonlinear associations between the IR indexes and AF recurrence after adjusting confounders that were statistically significant, with the reference point using the cut-off value of ROC for AF recurrence.

**Table 1:** Baseline characteristics of participants by recurrence following ablation

Variables	All(*n* = 2242)	Non-recurrence (*n* = 1531)	Recurrence (*n* = 711)	P value
**Clinical characteristics**				
Age, years	60.74 ± 11.29	60.35 ± 11.27	61.57 ± 11.30	0.017
Female, n (%)	821 (36.62)	538 (35.14)	283 (39.80)	0.033
BMI, kg/m^2^	25.06 ± 3.33	24.66 ± 3.12	25.94 ± 3.59	< 0.001
Current smoking, n (%)	445 (19.85)	296 (19.33)	149 (20.95)	0.370
Current drinking, n (%)	480 (21.41)	306 (19.99)	174 (24.47)	0.016
Hypertension, n (%)	1156 (51.56)	780 (50.95)	376 (52.88)	0.393
Diabetes mellitus, n (%)	631 (28.14)	399 (26.06)	232 (32.63)	0.001
Hyperlipidemia, n (%)	443 (19.76)	282 (18.42)	161 (22.64)	0.019
CHD, n (%)	675 (30.11)	440 (28.74)	235 (33.05)	0.038
Heart failure, n (%)	311 (13.87)	215 (14.04)	96 (13.50)	0.730
Prior stroke/TIA, n (%)	371 (16.55)	241 (15.74)	130 (18.28)	0.132
Duration of AF (≥ 24 months), n (%)	1141 (50.89)	728 (47.55)	413 (58.09)	< 0.001
AF type				< 0.001
Paroxysmal AF, n (%)	1305 (58.21)	950 (62.05)	355 (49.93)	
Persistent AF, n (%)	937 (41.79)	581 (37.95)	356 (50.07)	
**Medication**				
ACEI/ARB, n (%)	905 (40.47)	604 (39.50)	301 (42.57)	0.169
AADs, n (%)	1866 (83.23)	1268 (82.82)	598 (84.11)	0.448
Statins, n (%)	721 (32.16)	483 (31.55)	238 (33.47)	0.364
**Laboratory test**				
WBC, 10^9^/l	6.32 ± 1.74	6.33 ± 1.74	6.28 ± 1.75	0.452
PLT, 10^9^/l	198.22 ± 55.23	200.18 ± 55.48	193.97 ± 54.56	0.013
UA, mmol/l	327.11 ± 96.52	325.68 ± 94.73	329.63 ± 99.44	0.367
Cr, µmol/l	76.97 ± 36.01	76.79 ± 32.39	77.35 ± 42.81	0.734
FBG, mmol/l	5.48 ± 1.67	5.42 ± 1.60	5.68 ± 1.91	0.002
Hb1AC, %	6.13 ± 1.00	6.10 ± 0.0.99	6.20 ± 1.05	0.025
TC, mmol/l	3.72 ± 0.90	3.72 ± 0.89	3.75 ± 0.93	0.422
TG, mmol/l	1.23 (0.93–1.65)	1.22 (0.92–1.63)	1.24 (0.93–1.73)	0.066
HDL-C, mmol/l	1.12 ± 0.31	1.13 ± 0.33	1.11 ± 0.27	0.082
LDL-C, mmol/l	2.12 ± 0.72	2.11 ± 0.71	2.15 ± 0.74	0.277
**Echocardiographic**				
LVEF, %	61.56 ± 6.73	61.60 ± 6.73	61.50 ± 6.75	0.751
LA diameter, mm	39.85 ± 6.14	38.76 ± 5.96	42.21 ± 5.85	< 0.001
LVEDD, mm	47.43 ± 4.86	47.30 ± 4.76	47.72 ± 5.06	0.061
**Extra PV LA ablation**				
Linear ablation, n (%)	1415 (63.11)	960 (62.70)	455 (63.99)	0.556
SVC isolation, n (%)	270 (12.04)	193 (12.61)	77 (10.83)	0.229
CHA_2_DS_2_-VASc score	2 (1–4)	2 (1–3)	2 (1–4)	0.001
TyG index	8.58 ± 0.54	8.55 ± 0.54	8.63 ± 0.54	0.002
TyG-BMI index	215.19 ± 33.02	211.07 ± 30.86	224.06 ± 35.68	< 0.001
TG/HDL-C ratio	2.64 (1.82–3.75)	2.58 (1.82–3.66)	2.73 (1.83-4.00)	0.067
METS-IR	38.75 ± 6.54	37.97 ± 6.13	40.42 ± 7.07	< 0.001

AADs, anti-arrhythmic drugs; ACEI, angiotensin-converting enzyme inhibitor; AF, atrial fibrillation; ARB, angiotensin receptor blocker; BMI, body mass index; CHD, coronary heart disease; Cr, creatinine; FBG, fasting blood glucose; HbA1c, glycosylated hemoglobin; HDL-C, high-density lipoprotein cholesterol; LA, left atrial; LDL-C, low-density lipoprotein cholesterol; LVEF, left ventricular ejection fraction; LVEDD, left ventricular end-diastolic diameter; METS-IR, metabolic score for insulin resistance; PLT, platelet; PV, pulmonary vein; SVC, superior vena cava; TC, total cholesterol; TG, triglyceride; TG/HDL-C, triglyceride to high-density lipoprotein cholesterol; TyG, triglyceride and glucose; TyG-BMI, triglyceride glucose-body mass index; UA,uric acid, WBT, white blood cell

Furthermore, we aimed to investigate whether IR indexes could enhance the predictive performance of the baseline risk model, which including age, sex, BMI, current drinking, DM, hyperlipidemia, CHD, duration of AF (≥ 24 months), AF type, FBG, HbA1c, LA diameter, PLT, CHA_2_DS_2_-VASc. To assess the incremental predictive performance of AF recurrence after introducing four IR indexes to the baseline risk model, various measures were used including the calculation of C statistic, continuous net reclassification improvement (NRI), and integrated discrimination improvement (IDI). The C statistic was calculated to represent the performance of each model using “Survival” R package. Both the continuous NRI and IDI were calculated using “survIDINRI” R package. Variables including age, sex, hypertension, DM, hyperlipidaemia, CHD, AF type, duration of AF, and statins were taken into account in subgroup analysis. Then the association between METS-IR or TyG-BMI index and AF recurrence was further explored in DM and duration of AF (≥ 24 months) patients. All statistical analyses in the present study were performed with SPSS 26.0 from IBM SPSS Inc and R 3.6.1 by the R Development Core Team in Vienna, Austria. All tests were 2-sided, and *P* < 0.05 was considered statistically significant.

## Results

### Baseline characteristics

**Fig. 1:**
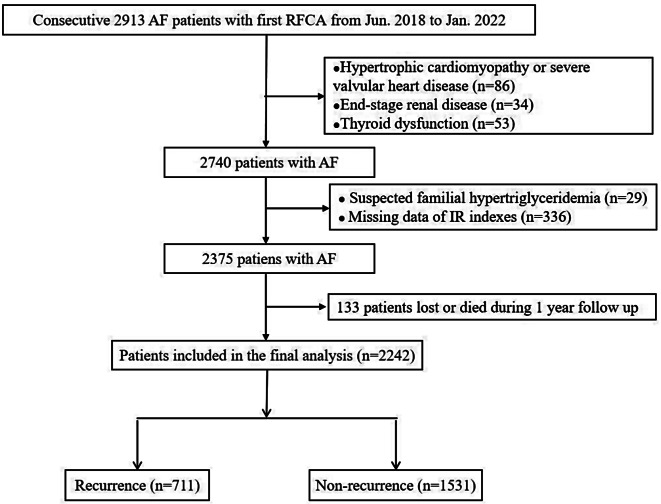
Flow diagram of patient’s selection. AF, atrial fibrillation; IR, insulin resistance

A total of 2931 patients with AF who underwent successful RFCA were screened for eligibility and 2242 patients with AF were included in the final analysis, as shown in Fig. 1. During a 1-year follow-up, a cumulative of 711 (31.71%) AF recurrence were documented. The comparison of baseline characteristics between patients with and without AF recurrence was shown in Table 1. Compared patients without AF recurrence, those with AF recurrence tended to be older and had a higher proportion of current drinking, CHD, hyperlipidemia, DM, PeAF, and duration of AF (≥ 24 months). They also had higher levels of BMI, FBG, Hb1AC, CHA_2_DS_2_-VASc, and LA diameter (*P* < 0.05). There was a lower level of platelets in recurrence patients, compared to those without recurrence (*P* < 0.05). Furthermore, patients in the recurrence group had significantly larger TyG index, METS-IR, and TyG-BMI index than those in the without recurrence group (*P* < 0.05). However, there was no significant difference in TG/HDL-C ratio between the two groups (*P* = 0.067).

### The predictive value of four IR indexes for AF recurrence

The ROC analysis was used to determine the optimal cut-of value of four indexes for predicting AF recurrence. The analysis determined that the optimal cut-off value for TyG index, METS-IR, TyG-BMI index, and TG/HDL-C ratio were 8.692 (sensitivity 45.1%, specificity 64.3%), 40.889 (sensitivity 44.6%, specificity 72.0%), 225.501 (sensitivity 46.1%, specificity 70.6%), and 3.272 (sensitivity 37.6%, specificity 68.1%). The area under the curve for four IR indexes were 0.539 (95% CI 0.513–0.565), 0.603 (95% CI 0.577–0.628), 0.608 (95% CI 0.583–0.633), 0.524 (95% CI 0.498–0.550); respectively (Additional file: Fig. S1). Additional file: Fig. S2 can be observed that Kaplan-Meier curves showed a noticeably increased risk of recurrence in patients belonging to the third IR indexes (TyG index, METS-IR, and TyG-BMI index) tertiles when compared to the remaining groups. However, no significant differences were noted in the cumulative 1-year incidence of AF recurrence between TG/HDL-C ratio tertiles groups. When IR indicators was used as a dichotomous indicator based on the optimal cut-off value of ROC for AF recurrence, Kaplan–Meier curves revealed that the cumulative 1-year incidence of AF recurrence was markedly higher in patients with high IR indexes than in those with low IR indexes (Additional file: Fig. S3).

### Relationship between TyG index and AF recurrence

**Fig. 2:**
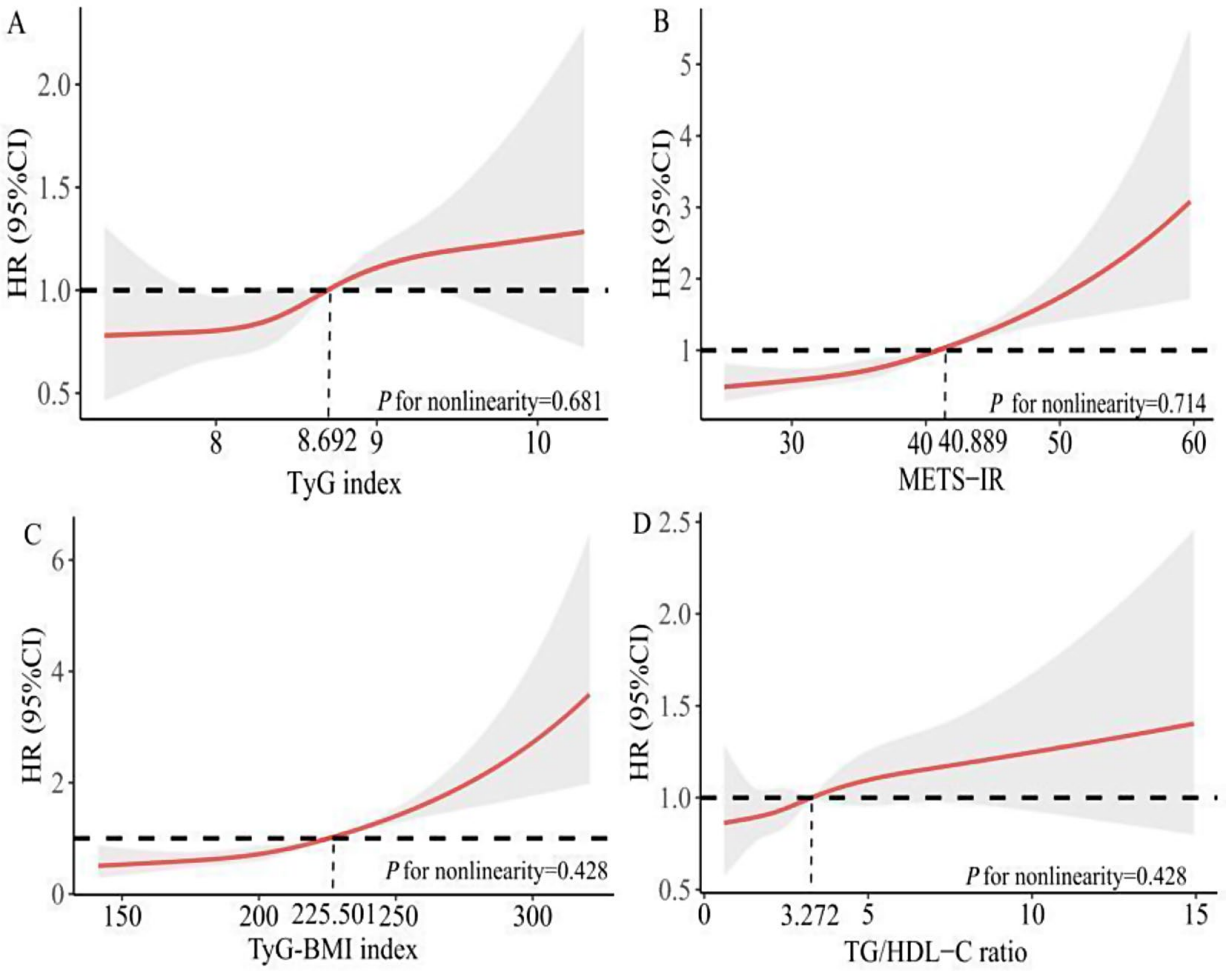
Restricted cubic spline curves for AF recurrence by four insulin resistance indexes after covariates adjustment. AF, atrial fibrillation; CI, confidence interval; HR, hazard ratio; METS-IR, metabolic score for insulin resistance; TyG, triglyceride and glucose; TyG-BMI, triglyceride glucose-body mass index; TG/HDL-C, triglyceride to high-density lipoprotein cholesterol

We observed the correlation between non-insulin-based IR indicators and AF recurrence following ablation from the perspectives of continuous and classified variables by adjusting for different risk factors. The first was not to adjust any risk factors; the second was to adjust age and gender; and the last was to adjust the risk factors screened by the univariate analysis. The results showed that the TyG index was significantly associated with AF recurrence regardless of whether as continuous variable or categorical variable by optimal cut-of value of ROC (*P* < 0.05). Then we grouped TyG index based on tertiles. The risk of AF recurrence was higher in the T3 group than in the T1 group, as shown in model 1 and model 2 (*P* < 0.05). The risk of AF recurrence for patients in T3 was 1.25 times greater (95% CI 1.03–1.51, *P* = 0.024) than in patients with T1 group after adjusting for age, sex, BMI, current drinking, DM, hyperlipidemia, CHD, duration of AF (≥ 24 months), AF type, HbA1c, LA diameter, PLT, CHA_2_DS_2_-VASc in the model 3 (Table 2). Moreover, the analysis of RCS revealed no apparent association was observed between the TyG index and the risk of AF recurrence after adjusted for clinical risk factors in the Model 3 (*P* for nonlinearity = 0.681), as shown in Fig. 2A.

**Table 2:** Association between TyG index (per 1 unit increase) and AF recurrence

	Model 1		Model 2		Model 3	
	HR (95%CI)	P value	HR (95% CI)	P value	HR (95% CI)	P value
TyG index	1.22 (1.07–1.39)	0.003	1.22 (1.07–1.40)	0.003	1.18 (1.02–1.36)	0.024
< 8.692	Reference		Reference		Reference	
≥ 8.692	1.37 (1.18–1.58)	< 0.001	1.37 (1.18–1.59)	< 0.001	1.40 (1.19–1.64)	< 0.001
T1	Reference		Reference		Reference	
T2	0.94 (0.78–1.14)	0.532	0.95 (0.78–1.14)	0.557	0.87 (0.71–1.05)	0.150
T3	1.29 (1.08–1.54)	0.005	1.30 (1.09–1.55)	0.004	1.25 (1.03–1.51)	0.024

AF, atrial fibrillation; CI, confidence interval; HR, hazard ratio; TyG, triglyceride and glucose; T1: TyG index ≤ 8.325; T2: 8.325 < TyG index ≤ 8.765; T3:8.765 < TyG index

Model 1: Unadjusted

Model 2: Adjusted for age and sex

Model 3: Adjusted for age, sex, body mass index, current drinking, diabetes mellitus,

hyperlipidemia, coronary heart disease, duration of AF (≥ 24 months), AF type, HbA1c, left atrial diameter, platelet, CHA_2_DS_2_-VASc score

### Relationship between METS-IR and AF recurrence

The METS-IR was an independent risk factor for AF recurrence whether as a continuous variable or categorical variable by optimal cut-of value of ROC (*P* < 0.05). The risk of AF recurrence was higher both in the M2 and M3 groups than in the M1 group, as shown in the model 4 and model 5 (*P* < 0.05). The risk of AF recurrence for patients in M3 was 1.82 times greater (95% CI 1.50–2.22, *P* < 0.001) than in patients with M1 group after adjusting for age, sex, current drinking, DM, hyperlipidemia, CHD, duration of AF (≥ 24 months), AF type, HbA1c, LA diameter, PLT, CHA_2_DS_2_-VASc in the model 6 **(**Table 3**)**. The RCS revealed a correlation between METS-IR and the risk of AF recurrence after adjusted for clinical risk factors in the Model 6 (*P* for nonlinearity = 0.714), as shown in Fig. 2B.

**Table 3:** Association between METS-IR (per 1 unit increase) and AF recurrence

	Model 4		Model 5		Model 6	
	HR (95%CI)	P value	HR (95% CI)	P value	HR (95% CI)	P value
METS-IR	1.04 (1.03–1.06)	< 0.001	1.04 (1.03–1.06)	< 0.001	1.04 (1.03–1.05)	< 0.001
< 40.889	Reference		Reference		Reference	
≥ 40.889	1.78 (1.54–2.07)	< 0.001	1.84 (1.58–2.13)	< 0.001	1.62 (1.38–1.89)	< 0.001
M1	Reference		Reference		Reference	
M2	1.32 (1.08–1.61)	0.007	1.34 (1.10–1.63)	0.004	1.26 (1.02–1.54)	0.030
M3	2.05 (1.71–2.47)	< 0.001	2.14 (1.77–2.58)	< 0.001	1.82 (1.50–2.22)	< 0.001

AF, atrial fibrillation; CI, confidence interval; HR, hazard ratio; METS-IR, metabolic score for insulin resistance; M1: METS-IR ≤ 35.585; M2: 35.585 < METS-IR ≤ 40.863; M3: 40.863 < METS-IR

Model 4: Unadjusted

Model 5: Adjusted for age and sex

Model 6: Adjusted for age, sex, current drinking, diabetes mellitus, hyperlipidemia, coronary heart disease, duration of AF (≥ 24 months), AF type, HbA1c, left atrial diameter, platelet, CHA_2_DS_2_-VASc score

### Relationship between TyG-BMI index and AF recurrence

Next, we found that TyG-BMI index was significantly associated with increased risk of AF recurrence regardless of whether TyG-BMI index as continuous variable or categorical variable by optimal cut-of value of ROC (*P* < 0.05). Then, TyG-BMI index was divided into three groups according to tertiles, with the B1 group as a reference, B3 group had a higher risk of AF recurrence in the model 7 and model 8 (*P* < 0.05). Compared with the B1 group in model 9 adjusted for age, sex, current drinking, DM, hyperlipidemia, CHD, duration of AF (≥ 24 months), AF type, HbA1c, LA diameter, PLT, CHA_2_DS_2_-VASc, B3 showed a 1.71 increased risk of AF recurrence (95% CI 1.41–2.07, *P* < 0.001), (Table 4). The RCS revealed a correlation between TyG-BMI index and the risk of AF recurrence after adjusted for clinical risk factors in the Model 9 (*P* for nonlinearity = 0.428), as shown in Fig. 2C.

**Table 4:** Association between TyG-BMI index (per 1 unit increase) and AF recurrence

	Model 7		Model 8		Model 9	
	HR (95%CI)	P value	HR (95% CI)	P value	HR (95% CI)	P value
TyG-BMI index	1.01 (1.01–1.01)	< 0.001	1.01 (1.01–1.01)	< 0.001	1.01 (1.01–1.01)	< 0.001
< 225.501	Reference		Reference		Reference	
≥ 225.501	1.76 (1.52–2.04)	< 0.001	1.79 (1.54–2.07)	< 0.001	1.55 (1.33–1.81)	< 0.001
B1	Reference		Reference		Reference	
B2	1.18 (0.97–1.44)	0.106	1.18 (0.97–1.44)	0.098	1.17 (0.96–1.44)	0.125
B3	1.93 (1.61–2.32)	< 0.001	1.97 (1.64–2.36)	< 0.001	1.71 (1.41–2.07)	< 0.001

AF, atrial fibrillation; CI, confidence interval; HR, hazard ratio; TyG-BMI, triglyceride glucose-body mass index; B1: TyG-BMI index ≤ 198.441; B2: 198.441 < TyG-BMI index ≤ 226.797; B3: 226.797 < TyG-BMI index

Model 7: Unadjusted

Model 8: Adjusted for age and sex

Model 9: Adjusted for age, sex, current drinking, diabetes mellitus, hyperlipidemia, coronary heart disease, duration of AF (≥ 24 months), AF type, HbA1c, left atrial diameter, platelet, CHA_2_DS_2_-VASc score

### Relationship between TG/HDL-C ratio and AF recurrence

TG/HDL-C ratio was not an independent risk factor for AF recurrence (*P* > 0.05). When the TG/HDL-C ratio was divided into two groups, in the model.

10, 11, and 12, the risk of AF recurrence was higher in TG/HDL-C ratio ≥ 3.272 group, compared with TG/HDL-C ratio < 3.272 group. Then, patients in group G3 had not a higher risk of AF recurrence (HR1.13, 95% CI 0.94–1.37, *P* = 0.195) in the model 12, compared with those in group G1 after adjusting for age, sex, BMI, current drinking, DM, hyperlipidemia, CHD, duration of AF (≥ 24 months), AF type, FBG, HbA1c, LA diameter, PLT, CHA_2_DS_2_-VASc (Table 5). Moreover, the analysis of RCS revealed no apparent association was observed between the TG/HDL-C ratio and risk of recurrence after adjusted for baseline clinical risk factors in the Model 12 (*P* for nonlinearity = 0.795), as shown in Fig. 2D.

**Table 5:** Association between TG/HDL-C ratio(per 1 unit increase) and AF recurrence

	Model 10		Model 11		Model 12	
	HR (95%CI)	P value	HR (95% CI)	P value	HR (95% CI)	P value
TG/HDL-C ratio	1.03 (1.00-1.06)	0.050	1.03 (1.01–1.06)	0.021	1.03 (1.00-1.06)	0.055
< 3.272	Reference		Reference		Reference	
≥ 3.272	1.22 (1.05–1.42)	0.010	1.26 (1.08–1.47)	0.003	1.22 (1.04–1.43)	0.017
G1	Reference		Reference		Reference	
G2	0.98 (0.81–1.18)	0.822	0.99 (0.82–1.19)	0.921	0.91 (0.75–1.10)	0.343
G3	1.19 (0.99–1.42)	0.059	1.23 (1.03–1.48)	0.022	1.13 (0.94–1.37)	0.195

AF, atrial fibrillation; CI, confidence interval; HR, hazard ratio; TG/HDL-C, triglyceride to high-density lipoprotein cholesterol; G1: TG/HDL-C ratio ≤ 2.097; G2: 2.097 < TG/HDL-C ratio ≤ 3.287; G3: 3.287 < TG/HDL-C ratio

Model 10: Unadjusted

Model 11: Adjusted for age and sex

Model 12: Adjusted for age, sex, body mass index, current drinking, diabetes mellitus, hyperlipidemia, coronary heart disease, duration of AF (≥ 24 months), AF type, fasting blood glucose, HbA1c, left atrial diameter, platelet, CHA_2_DS_2_-VASc score

### Non-insulin-based IR indexes incremental predictive value

The predictive value of non-insulin-based IR indexes for AF recurrence was shown in Table 6. The calibration plots for the basic risk model predicting AF recurrence exhibited excellent agreement between the observed and predicted probabilities (Additional file: Fig. S4). When incorporating METS-IR or TyG-BMI index into the baseline risk model, a significant improvement in the predictive ability for recurrence was noted, as evidenced by the C-statistic rising from 0.690 to 0.701 (*P* = 0.047) and 0.702 (*P* = 0.045), respectively. Significant enhancements in the NRI and IDI were also observed as a result of incorporating the METS-IR and TyG-BMI index into the baseline risk model (*P* < 0.05). Unfortunately, the results showed no significant incremental predictive ability of TyG index and TG/HDL-C ratio to the baseline risk model (*P* > 0.05). Furthermore, we compared the AUC area of METS and TyG-BMI index with the traditional risk factors (type of AF, BMI and LA diameter) for AF recurrence. The AUC area of METS-IR (0.603) and TyG-BMI index (0.608) were larger than AF type (0.561) and BMI (0.533). However, LA diameter (0.670) had a larger AUC area for AF recurrence, compared to the METS-IR and TyG-BMI index, (Additional file: Fig. S5).

**Table 6:** Added predictive ability and reclassification statistics of insulin resistance

	C-statistic (95% CI)	P value	IDI (95% CI)	P value	Continuous NRI (95% CI)	P value
Baseline risk model	0.690 (0.667–0.714)	Reference	Reference		Reference	
+TyG index	0.691 (0.667–0.714)	0.855	0.003 (-0.004-0.012)	0.432	0.042 (-0.034-0.109)	0.334
+METS-IR	0.701 (0.677–0.724)	0.047	0.020 (0.008–0.034)	0.004	0.119 (0.056–0.170)	0.002
+TyG-BMI index	0.702 (0678-0.725)	0.045	0.024 (0.012–0.038)	< 0.001	0.134 (0.079–0.182)	< 0.001
+TG/HDL-C ratio	0.692 (0.669–0.716)	0.455	0.001 (-0.005-0.006)	0.819	-0.001 (-0.059-0.079)	0.429

Baseline risk model: age, sex, body mass index, current drinking, diabetes mellitus, hyperlipidemia, coronary heart disease, duration of AF (≥ 24 months), AF type, fasting blood glucose, HbA1c, left atrial diameter, platelet, CHA_2_DS_2_-VASc score

AF, atrial fibrillation; CI, confidence interval; IDI, integrated discrimination improvement; METS-IR, metabolic score for insulin resistance; NRI, net reclassification improvement; TyG, triglyceride and glucose; TyG-BMI, triglyceride glucose-body mass index; TG/HDL-C, triglyceride to high-density lipoprotein cholesterol

**Fig. 3:**
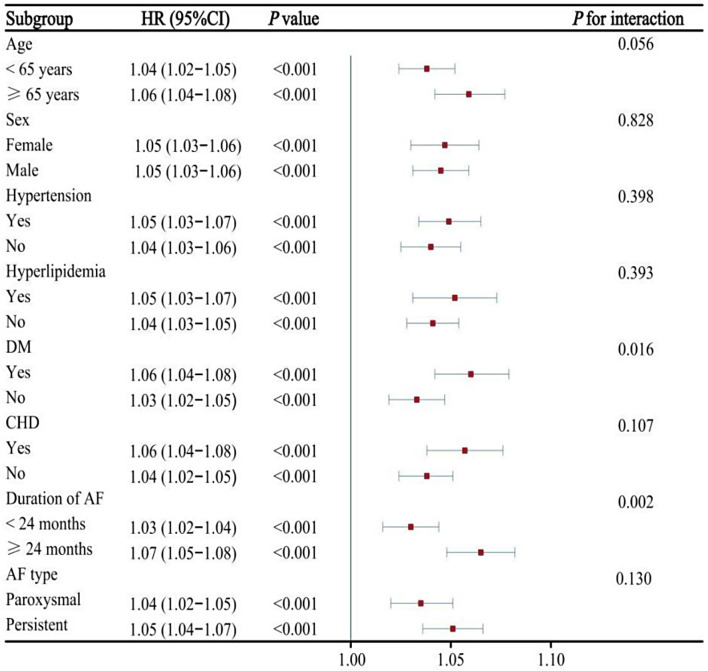
Association between METS-IR (per 1 unit increase) and AF recurrence following ablation in different subgroups. AF, atrial fibrillation; CHD, coronary heart disease; DM,diabetes mellitus; METS-IR, metabolic score for insulin resistance

### Subgroup analysis

**Fig. 4:**
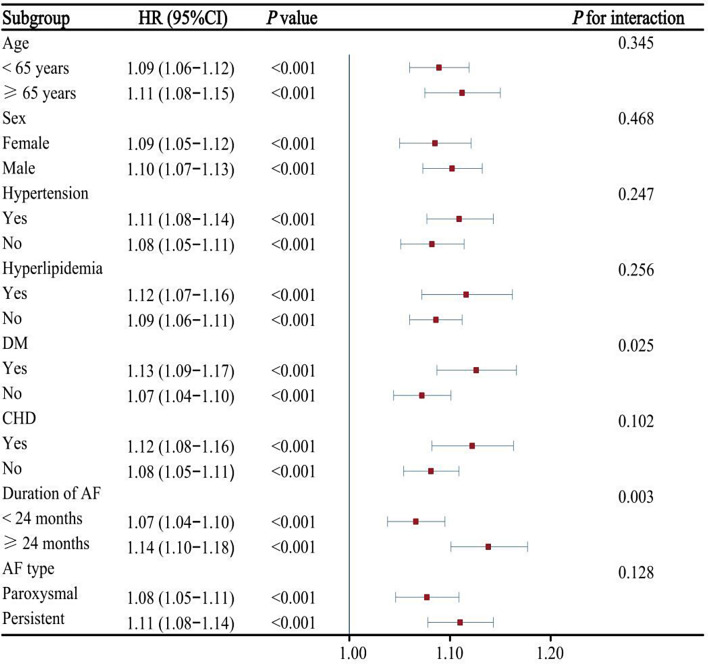
Association between TyG-BMI index (per 10 unit increase) and AF recurrence following ablation in different subgroups. AF, atrial fibrillation; CHD, coronary heart disease; DM,diabetes mellitus; TyG-BMI, triglyceride glucose-body mass index

Next, we conducted the subgroups analysis stratified by age, sex, hypertension, DM, hyperlipidemia, CHD, AF type, and duration of AF. As shown in Figs. 3 and 4. METS-IR and TyG-BMI index were associated with AF recurrence in different subgroups. Moreover, the results indicated a significant interaction between DM subgroups and METS-IR or TyG-BMI index on the risk of AF recurrence (*P* for interaction < 0.05). Similarly, significant interactions were detected between duration of AF (≥ 24 months) and either METS-IR or TyG-BMI index (*P* for interaction < 0.05). However, no significant interaction was observed in the subgroup analysis of TyG index or TG/HDL-C ratio with AF recurrence (*P* > 0.05), as shown in Additional file: Figs. S6 and S7. Importantly, we did not find a significant interaction between four indicators of IR and AF recurrence based on whether using statins medication on admission (Additional file: Table S1).

**Fig. 5:**
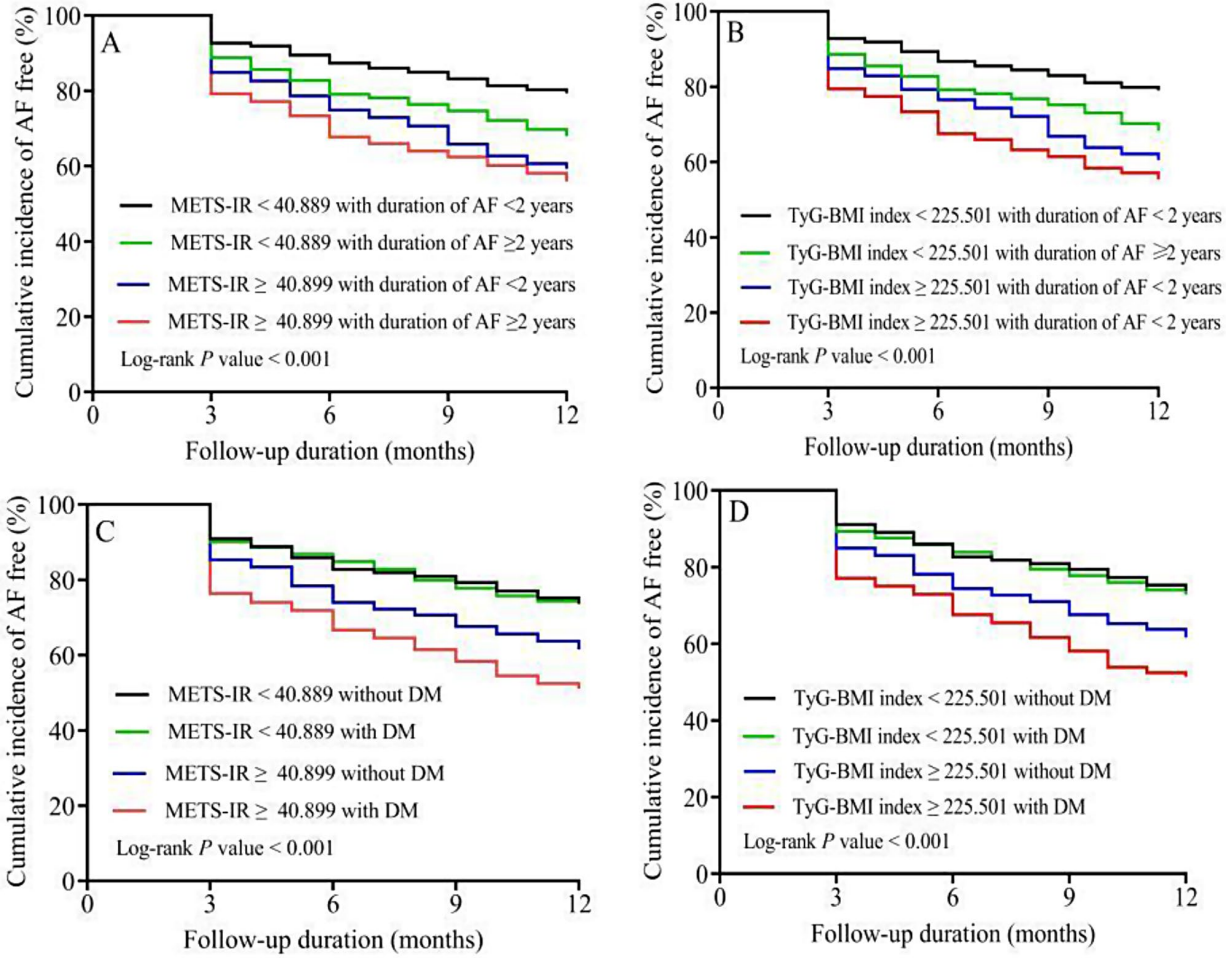
Kaplan-Meier estimated event rates of AF recurrence following ablation according to cut-off values of ROC curves for insulin resistance indexes and AF duration or diabetes mellitus. AF, atrial fibrillation; METS-IR, metabolic score for insulin resistance; ROC, receiver operating curve; TyG-BMI, triglyceride glucose-body mass index

### Association of METS-IR or TyG-BMI index with AF reucrrence in DM patients

When all study patients were divided into 4 groups based on a combination of DM (DM and without DM) and METS-IR or TyG-BMI index (METS-IR ≥ 40.899 and < 40.899, or TyG-BMI index ≥ 225.501 and < 225.501), patients with both DM and high METS-IR or TyG-BMI index had a markedly higher cumulative incidence of AF recurrence than the remaining patients (Fig. 5A and B). After adjusting for age, sex, current drinking, hyperlipidemia, CHD, duration of AF (≥ 24 months), AF type, HbA1c, LA diameter, PLT, and CHA_2_DS_2_-VASc. METS-IR ≥ 40.899 or TyG-BMI index ≥ 225.501 with or without DM were independent risk factors for AF recurrence, as shown in Additional file: Table S2. According to METS-IR and TyG-BMI index cut-off value of ROC for AF recurrence in DM and non-DM populations. The RCS also revealed a correlation between METS-IR or TyG-BMI index and the risk of AF recurrence after adjusted for risk factors in DM and non-DM patients, as shown in Fig. S8. When incorporating METS-IR or TyG-BMI index into the baseline risk model, a significant improvement in the predictive ability for recurrence was noted by the C-statistic, NRI, and IDI in DM patients (*P* < 0.05). In the non-DM population, we did not find significant improvements in C-index, IDI, and NRI with the addition of TyG-BMI or METS-IR to the basic risk model (*P* > 0.05), (Additional file: Table S3.

### Association of METS-IR or TyG-BMI index with AF recurrence in duration of AF (≥ 24 months) patients

Furthermore, when patients were divided into 4 groups based on a combination of AF duration (duration of AF ≥ 24 months and < 24 months) and METS-IR or TyG-BMI index (METS-IR ≥ 40.899 and < 40.899, or TyG-BMI index ≥ 225.501 and < 225.501), patients with both duration of AF ≥ 24 months and high METS-IR or TyG-BMI index had a markedly higher cumulative incidence of AF recurrence than the remaining patients (Fig. 5C and D). After adjusting for age, sex, current drinking, hyperlipidemia, CHD, DM, AF type, HbA1c, LA diameter, PLT, and CHA_2_DS_2_-VASc. METS-IR ≥ 40.899 or TyG-BMI index ≥ 225.501 with duration of AF ≥ 24 months were independent risk factor for AF recurrence after ablation (HR 2.18, 95% CI 1.74–2.74, *P* < 0.001, or HR 2.10, 95% CI 1.68–2.63, *P* < 0.001; respectively), as shown in Additional file: Table S4. Based on the METS-IR and TyG-BMI index cut-off values of ROC for AF recurrence in duration of AF ≥ 24 and < 24 months patients, the analysis of RCS in duration of AF ≥ 24 and < 24 months patients are shown in Fig. S9. The improvement in model prediction accuracy when incorporating METS-IR or TyG-BMI index into the baseline risk model in the duration of AF ≥ 24 and < 24 months patients, as shown in Additional file: Table S5.

## Discussion

In the present study, we provide the first report of the relationship between TyG-BMI index and AF recurrence after ablation. Notably, this is also the first study comparing the value of TyG index, METS-IR, TyG-BMI index, and TG/HDL-C ratio in predicting AF recurrence. A larger TyG index, METS-IR, and TyG-BMI index were associated with an elevated risk of AF recurrence. The integration of METS-IR or TyG-BMI index into the baseline risk model may have improved the accuracy in predicting the risk of AF recurrence, however absolute increase in C-statistic is not clinically meaningful. Co-presence of DM or long duration of AF and high TyG-BMI index or METS-IR portends a high risk for AF recurrence. By addressing this research gap, our findings may exert far-reaching significance for follow-up strategy of AF ablation patients.

IR, a pivotal hormone that regulates cellular metabolism, is closely linked to the progression of metabolism disorders [18]. IR has also been demonstrated to be closely related to the occurrence and prognosis of atherosclerotic cardiovascular disease, regardless of the presence of DM [19]. Previous study indicated that TyG index is a valuable tool for predicting the risk of all-cause and cardiovascular disease mortality in cardiovascular disease patients with diabetes or pre-diabetes [20]. Recent study results strongly suggested that TyG index may be used as a useful risk predictor to assess the long-term outcomes in acute ischemic stroke patients with DM [21]. Previous studies have found that TyG index, METS-IR, TyG-BMI index, and TG/HDL-C ratio are not only valid indicators for assessing IR, but also may be associated with cardiovascular diseases [22, 23]. A study meta-analysis showed that higher levels of IR were associated with higher risk of heart failure after adjusting other risk factors. This association was present in the study including diabetic and non-diabetic patients [24]. Recent study revealed that elevated TyG index is associated with an increased risk of stroke in hypertensive patients [25]. Previous study also suggested that IR evaluated with homeostasis model assessment-IR index is independently associated with the presence of diffusion-weighted imaging lesions in patients with acute and subacute primary intracerebral hemorrhage [26]. Moreover, one study found a higher incidence of AF in hospitalized patients with an elevated TyG index. After correction for traditional risk factors for AF, TyG index remained an independent risk factor for AF [27]. Another study revealed that homeostasis model assessment of IR was independent risk factors for AF recurrence after RFCA in AF patients without diabetes [28]. Additional, recurrence of AF after ablation was higher in diabetic patients than in non-diabetic patients, and metabolic abnormalities in diabetic patients have a key role in promoting arrhythmias [29]. However, data from the Framingham Heart Study fail to show relationship between IR and AF events [30]. In the present study, we first evaluated whether non-insulin-based IR related indicators could predict AF recurrence following ablation.

Results of the present study showed that AF patients in the AF recurrence group still had significantly higher TyG index, METS-IR, and TyG-BMI index than those in the non-recurrence group after adjusting for confounding factors. Previous studies have shown that TyG-BMI index is associated with the development of coronary atherosclerosis and increased risk of ischemic stroke [31–33]. Our study revealed that TyG-BMI index was not only an independent risk factor for AF recurrence but also had the highest predictive value, compared with TyG index, METS-IR, and TG/HDL-C ratio. Evidence from the Korean population suggested that TyG-BMI index is superior to TyG index in predicting IR, which is consistent with the present study. Bello-Chavolla et al. introduced METS-IR as a promising new metric to assess cardio-metabolism risk and screen for insulin sensitivity [34]. To date, no studies have investigated the correlation between METS-IR and recurrence after AF ablation. Notably, this study demonstrated that METS-IR could predict AF recurrence, with the higher predictive value compared with TyG index and TG/HDL-C ratio. Recent research showed that an elevated pre-ablation TyG index is associated with an increased risk of AF recurrence after RFCA in non-diabetic patients [35]. We demonstrated that TyG index was an independent risk factor for recurrence after AF ablation, both in diabetic and non-diabetic patients. However, we revealed that TG/HDL-C ratio was not an independent predictor for AF recurrence after adjusting for other risk factors. Wu et al. [36] also confirmed that TG/HDL-C ratio was not an independent predictor for the existence of CHD.

Recently, METS-IR and TyG-BMI have been recognized as a more reliable indicators for assessing IR [33, 37]. In our study, METS-IR and TyG-BMI index had a larger AUC area for AF recurrence after ablation, compared to traditional risk factor BMI and AF type, although LA diameter had a larger AUC area than METS-IR and TyG-BMI index. The discovery in our study was improved in forecast precision for AF recurrence when integrating the METS-IR or TyG-BMI index into the baseline risk model. Furthermore, METS-IR or TyG-BMI index showed a significant interaction for AF recurrence in both DM and long duration of AF (≥ 24 months) patients. DM may increase the size of LA or impair left ventricular diastolic function, accompanied by more co-morbidities, thereby increasing susceptibility to AF [38]. Diabetes was usually accompanied by severe IR, compared with non-diabetes patients [39, 40]. Co-presence of DM and high TyG-BMI index or METS-IR portends a high recurrence risk of AF. In addition, we also found that TyG-BMI index and METS-IR were significantly higher in individuals with recurrence compared to those without recurrence, regardless of diabetes status. Moreover, Previous study demonstrated that the long duration of AF was an independent factor for recurrence following ablation [41]. A possible explanation for the relationship between AF duration and recurrence is a vicious circle. Long AF duration prior to ablation, was often considered a surrogate marker for increased AF burden and enhanced atrial remodeling, predicting a poorer prognosis [42]. We considered that the coexistence of IR and a long course of AF could promote metabolic disorders, which also suggested that early rhythm intervention might reduce late recurrence of AF. Therefore, long duration of AF or DM with high IR indexes should be recognized as a high-risk for recurrence and require an intensive follow-up after ablation. By conducting research in different groups from different angles, we can have a deeper understanding of non-insulin-based IR indicators, selecting more valuable one for clinical application. Further investigations are urgently required to validate these findings.

A state of IR leads to an excessive accumulation of lipids in cardiomyocytes. This accumulation results in a phenomenon known as “cardiac lipotoxicity”. Cardiac lipotoxicity induces cellular dysfunction, cardiomyocyte apoptosis and impaired myocardial metabolism. These changes may cause functional and structural alterations in cardiomyocytes, thereby increasing the risk of AF [43]. IR increases CaMKIIδ oxidation and phosphorylation levels of phosphatidylban/RyR2, which leading to abnormal intracellular calcium homeostasis and atrial structural remodelling [44]. Animal studies have also found that IR induces impaired transport and expression of the major myocardial isoform of glucose transporter protein (GLUT) 4 and the novel subtype GLUT8, which increases the susceptibility to AF. IR was associated with multiple aspects of LA remodeling, including increased oxidative stress damage, elevated expression of hyperphosphorylated calc-related proteins, and cardiac fibrosis [45]. It has been shown that IR induces structural remodelling of the atria and abnormal intracellular calcium homeostasis [46]. Increased oxidative stress and inflammation were associated with impaired IR and insulin secretion, resulting in atrial electrical remodeling, LA fibrosis and LA low-pressure area, which may be the main components of the pathophysiology of AF [35, 47]. Most components of IR, such as abnormal glucose tolerance, obesity, and abnormal lipid levels, have been found to have a cumulative effect on the risk of AF progression, which may be associated with AF recurrence after RFCA.

This study still had some limitations. First, the study had a retrospective nature, and selection bias could not be avoided. Second, when assessing the LA size, measuring LA volume instead of LA diameter is recommended. LA volume was not routinely measured in the present study. Third, some patients with AF recurrence might have been missed for asymptomatic arrhythmia, although we encouraged patients to contact the physician in the case of suspected recurrence symptoms. Our study follow-up was also based on 12-lead electrocardiogram or Holter monitoring. Patch-type ECG, patient-triggered detection devices, or implantable loop recorders were not used in this study, which may lead to missed diagnosis in patients with AF recurrence. In addition, despite adjusting for many potential confounders, there may still be unmeasured confounders that could influence the observed associations. Information on other glucose related factors, diabetes medications, and insulin levels, were not available. However, we excluded some factors that are closely related to drug use, such as HbA1c and DM. This might weaken the effect of the drug on the results. Our study did not further collect low voltage area of LA, so the relationship between IR and low voltage area of LA needs to be further evaluated. Furthermore, although we validated our findings using different statistical analysis and multiple Cox models. Non-insulin-based indicators of IR may be co-linear with hyperlipidemia, DM and other indicators in multivariate Cox models, leading to biased results of statistical analysis. Finally, our study was retrospective and did not collect complications after AF ablation to explore the correlation between IR and complications. To address these limitations, future prospective studies must include larger sample sizes and more extensive data to confirm our findings.

## Conclusions

In summary, TyG index, METS-IR and TyG-BMI index were independently associated with AF recurrence following ablation. Non-insulin-based IR indexes may be useful for recurrence risk stratification for AF ablation. Comparison of the four non-insulin-based IR indexes showed that the TyG-BMI index had the highest predictive value for AF recurrence, followed by METS-IR. Co-presence of DM or long duration of AF and high TyG-BMI index or METS-IR portends a dismal prognosis. Our findings support the utility of non-insulin-based IR indexes as a reliable indicator for AF recurrence risk stratification in the real world. More studies are necessary to investigate if interventions that aim to address IR, as evaluated by non-insulin-based IR indexes, can improve the clinical outcomes for patients with AF ablation.

### Electronic supplementary material

Below is the link to the electronic supplementary material.

**Additional file: Figure S1. ** Receiver operating curve for the use of four insulin resistance indexes in the detection of AF recurrence following ablation. **Figure S2.** Kaplan–Meier estimated event rates of AF recurrence following ablation according to tertiles of insulin resistance indexes. **Figure S3.** Kapla-Meier estimated event rates of AF recurrence following ablation according to cut-off values of ROC curves for insulin resistance indexes **Figure S4.** The calibration plots for the adjusted model predicting AF recurrence. **Figure S5.** Receiver operating curve for risk factors in the detection of AF recurrence following ablation. **Figure S6.** Association between TyG index (per 1 unit increase) and AF recurrence following ablation in different subgroups. **Figure S7.** Association between TG/HDL-C ratio (per 1 unit increase) and AF recurrence following ablation in different subgroups. **Figure S8.** Restricted cubic spline curves for AF recurrence by METS-IR ans TyG-BMI index after covariate adjustment in DM and non-DM patients. **Figure S9.** Restricted cubic spline curves for AF recurrence by METS-IR and TyG-BMI index after covariates adjustment in duration of AF ≥ 24 and < 24 months patients. **Additional file: Table S1.** Association between non-insulin-based IR indexes and AF recurrence after ablation stratified by the statins medication at admission. **Table S2. **Association between METS-IR or TyG-BMI index with DM or non-DM and AF recurrence after ablation. **Table S3.** Added predictive ability and reclassification statistics of METS-IR and TyG-BMI index in DM and non-DM patients. **Table S4.** Association between METS-IR or TyG-BMI index with duration of AF ≥ 24 or < 24months and AF recurrence after ablation. **Table S5.** Added predictive ability and reclassification statistics of METS-IR and TyG-BMI index in duration of AF ≥ 24 and < 24 months patients.

## Data Availability

No datasets were generated or analysed during the current study.

## Abbreviations

AF: Atrial fibrillation
BMI: Body mass index
CHD: Coronary heart disease
CI: Confidence interval
CPVI: Circumferential pulmonary vein isolation
Cr: Creatinine
DM: Diabetes mellitus
FBG: Fasting blood glucose
HbA1c: Glycated haemoglobin
HR: Hazard ratio
HDL-C: High-density lipoprotein cholesterol
IDI: Integrated discrimination improvement
IR: Insulin resistance
LA: Left atrial
LDL-C: Low-density lipoprotein cholesterol
METS-IR: Metabolic score for insulin resistance
NRI: Net reclassification improvement
PAF: Paroxysmal atrial fibrillation
PeAF: Persistent atrial fibrillation
PLT: Platelet
RFCA: Radiofrequency catheter ablation
RCS: Restricted cubic spline
ROC: Receiver operating characteristic
SVC: Superior vena cava
TC: Total cholesterol
TyG: Triglyceride and glucose
TyG-BMI: Triglyceride glucose-body mass index
TG: Triglyceride
TG/HDL-C: Triglyceride to high-density lipoprotein cholesterol
UA: Uric acid
