# Supplementary material for: Non-insulin-based insulin resistance indexes in predicting atrial fibrillation recurrence following ablation: a retrospective study
Source: Cardiovasc Diabetol. 2024 Feb 28;23:87. doi: 10.1186/s12933-024-02158-6 (PMC10902970; doi:10.1186/s12933-024-02158-6)
Supplement: Supplementary file 1 — Additional file: Figure S1. Receiver operating curve for the use of four insulin resistance indexes in the detection of AF recurrence following ablation. Figure S2. Kaplan–Meier estimated event rates of AF recurrence following ablation according to tertiles of insulin resistance indexes. Figure S3. Kapla-Meier estimated event rates of AF recurrence following ablation according to cut-off values of ROC curves for insulin resistance indexes Figure S4. The calibration plots for the adjusted model predicting AF recurrence. Figure S5. Receiver operating curve for risk factors in the detection of AF recurrence following ablation. Figure S6. Association between TyG index (per 1 unit increase) and AF recurrence following ablation in different subgroups. Figure S7. Association between TG/HDL-C ratio (per 1 unit increase) and AF recurrence following ablation in different subgroups. Figure S8. Restricted cubic spline curves for AF recurrence by METS-IR ans TyG-BMI index after covariate adjustment in DM and non-DM patients. Figure S9. Restricted cubic spline curves for AF recurrence by METS-IR and TyG-BMI index after covariates adjustment in duration of AF ≥ 24 and < 24 months patients. Additional file: Table S1. Association between non-insulin-based IR indexes and AF recurrence after ablation stratified by the statins medication at admission. Table S2. Association between METS-IR or TyG-BMI index with DM or non-DM and AF recurrence after ablation. Table S3. Added predictive ability and reclassification statistics of METS-IR and TyG-BMI index in DM and non-DM patients. Table S4. Association between METS-IR or TyG-BMI index with duration of AF ≥ 24 or < 24months and AF recurrence after ablation. Table S5. Added predictive ability and reclassification statistics of METS-IR and TyG-BMI index in duration of AF ≥ 24 and < 24 months patients. [file 12933_2024_2158_MOESM1_ESM.docx]

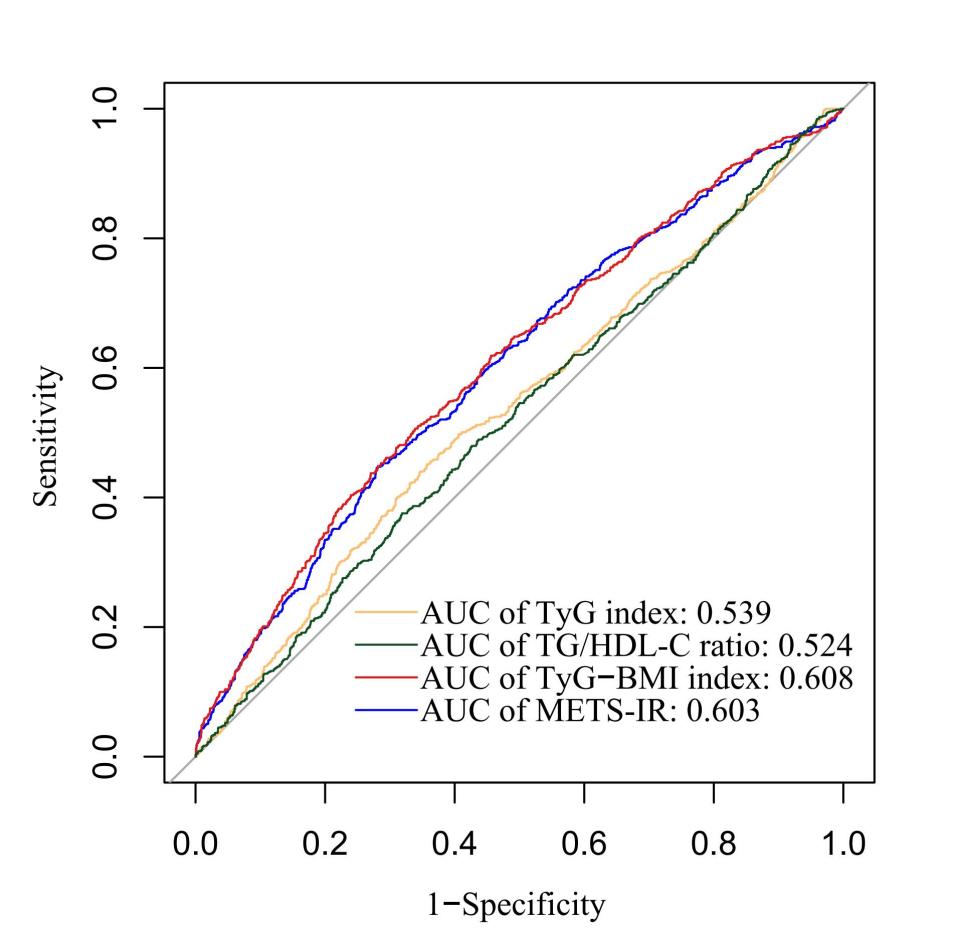


**Figure. S1** Receiver operating curve for the use of four insulin resistance indexes in the detection of AF recurrence following ablation. AUC, area under curve; METS-IR, metabolic score for insulin resistance; TyG, triglyceride and glucose; TyG-BMI, triglyceride glucose-body mass index; TG/HDL-C, triglyceride to high-density lipoprotein cholesterol


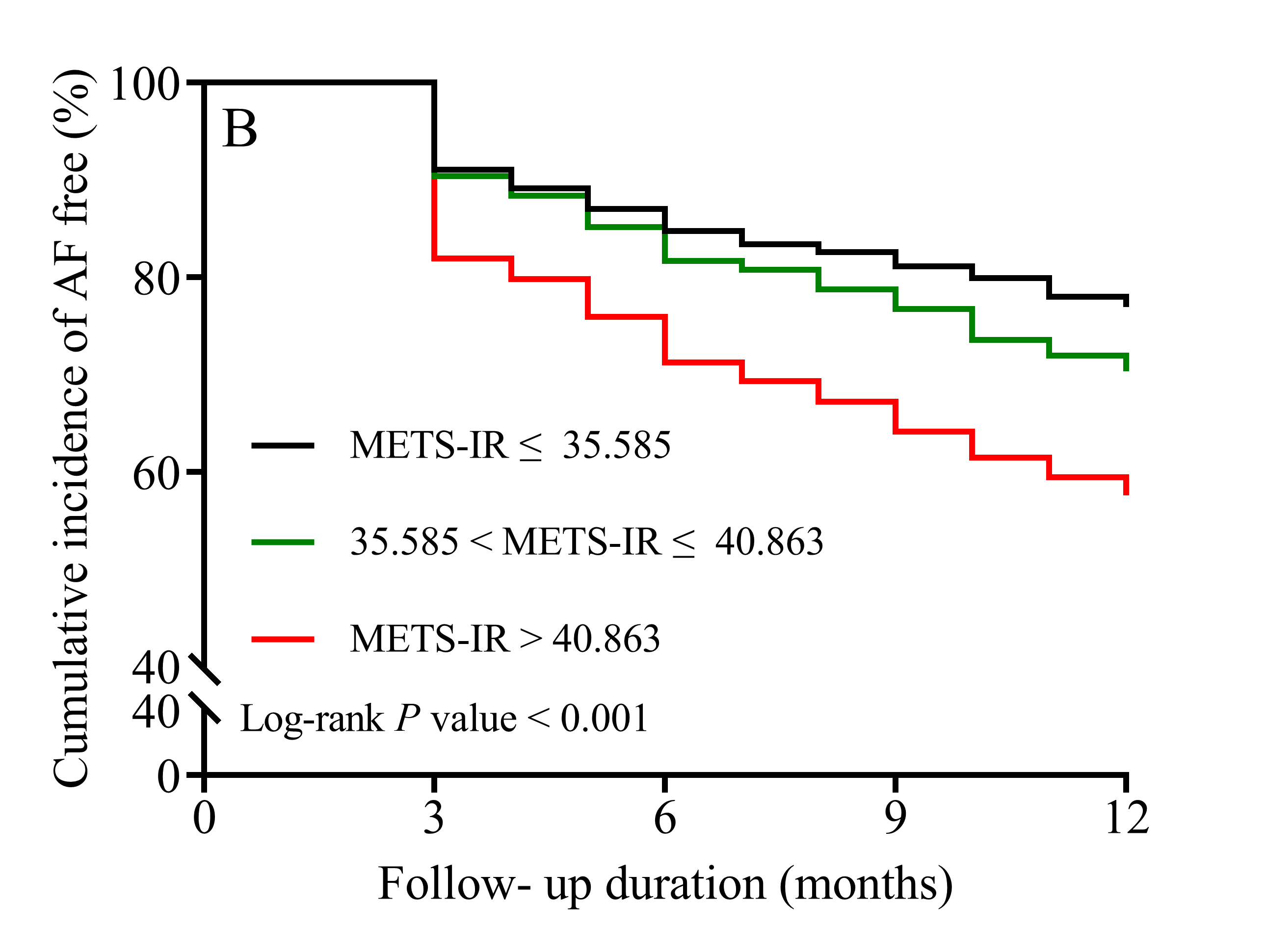

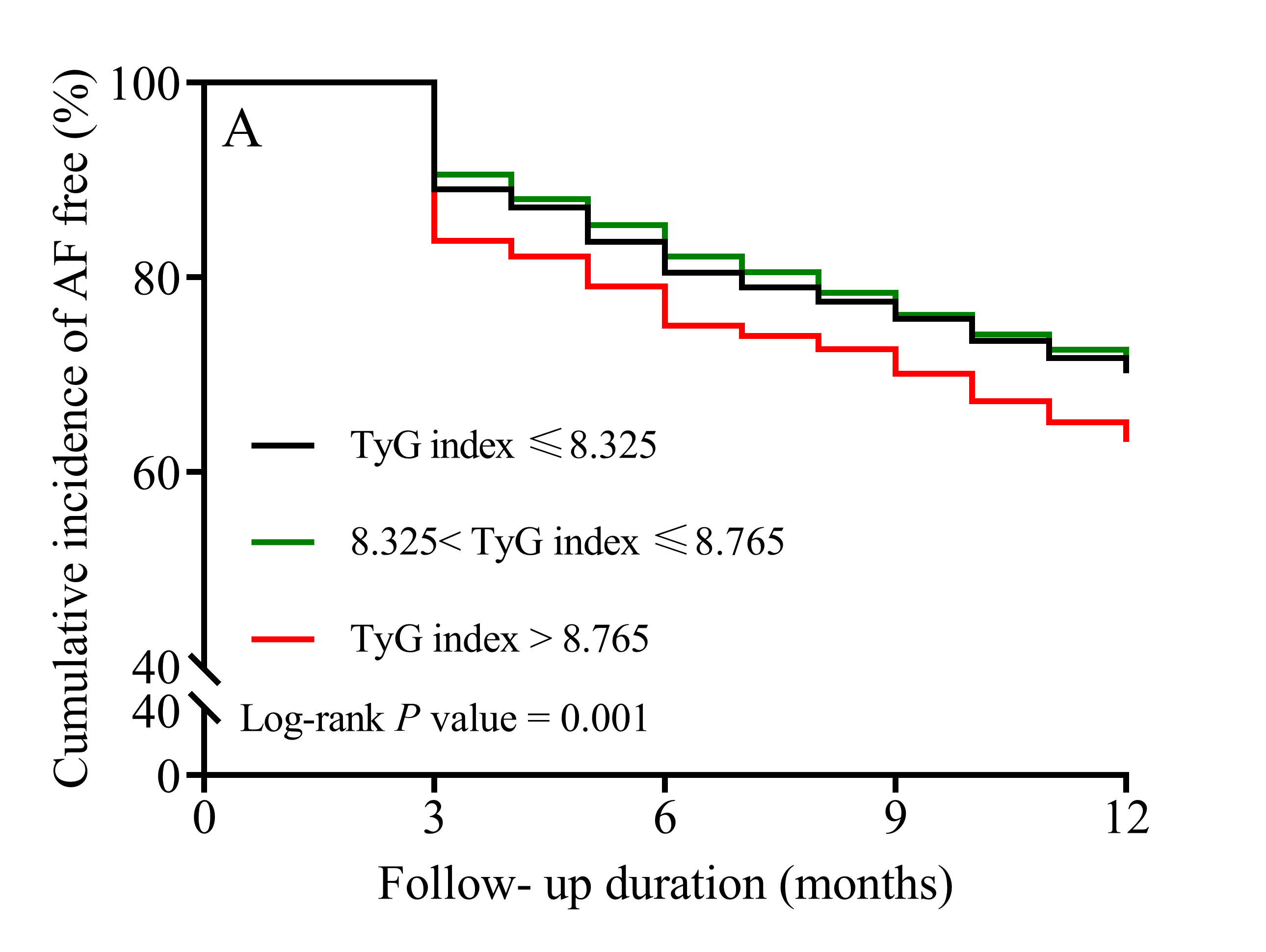


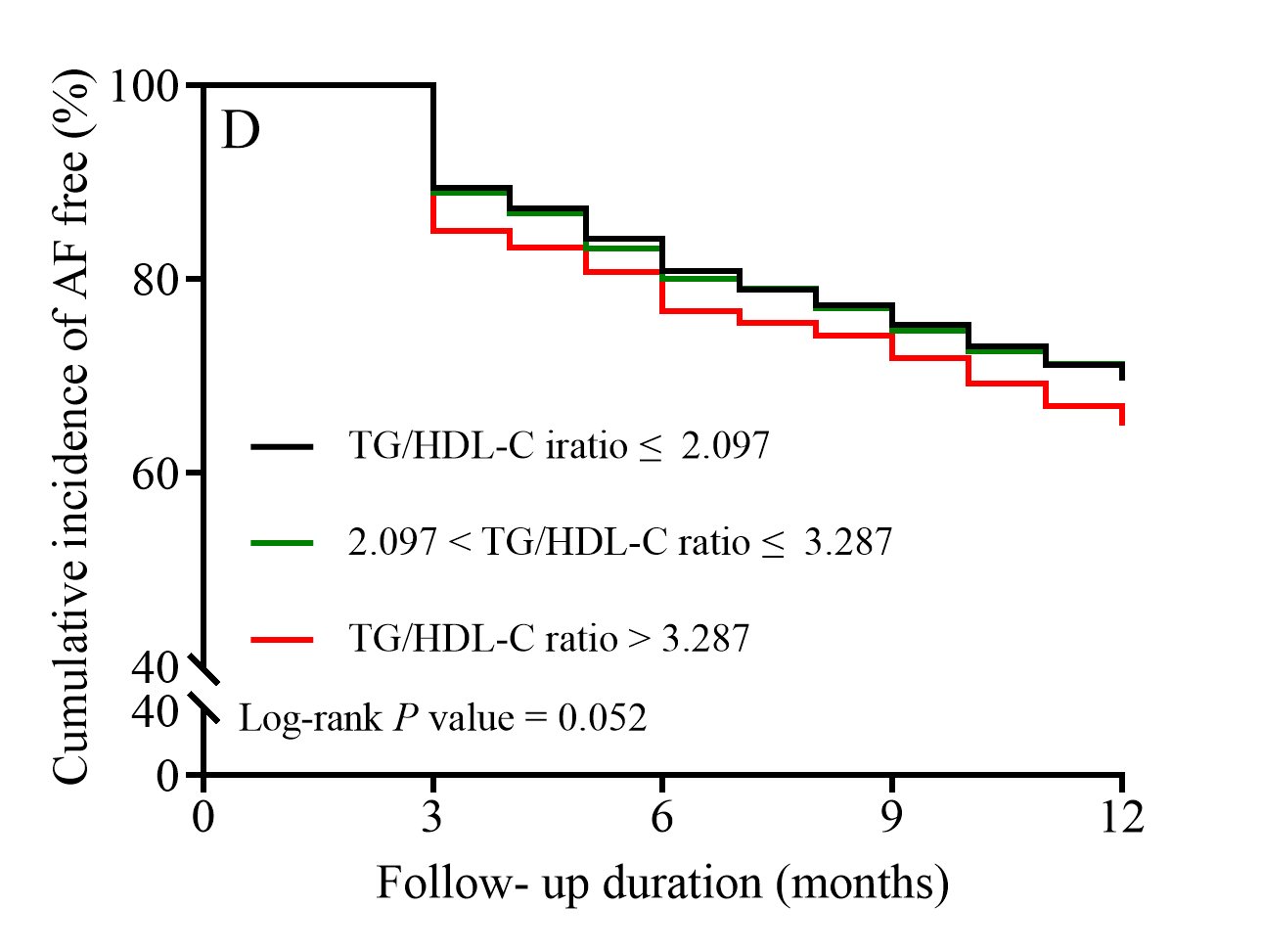

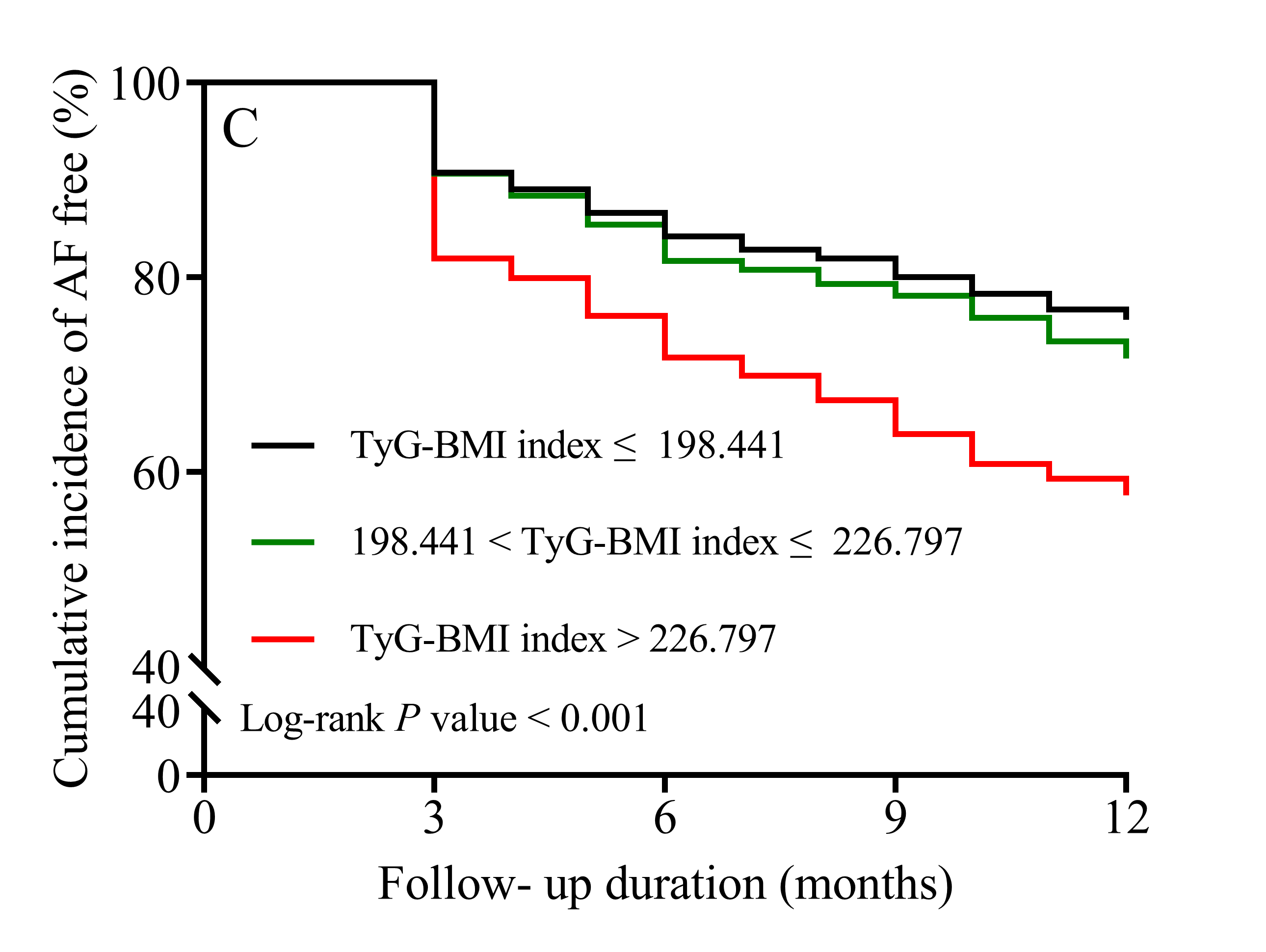
**Fig. S2** Kaplan–Meier estimated event rates of AF recurrence following ablation according to tertiles of insulin resistance indexes. AF, atrial fibrillation; METS-IR, metabolic score for insulin resistance; TyG, triglyceride and glucose; TyG-BMI, triglyceride glucose-body mass index; TG/HDL-C, triglyceride to high-density lipoprotein cholesterol


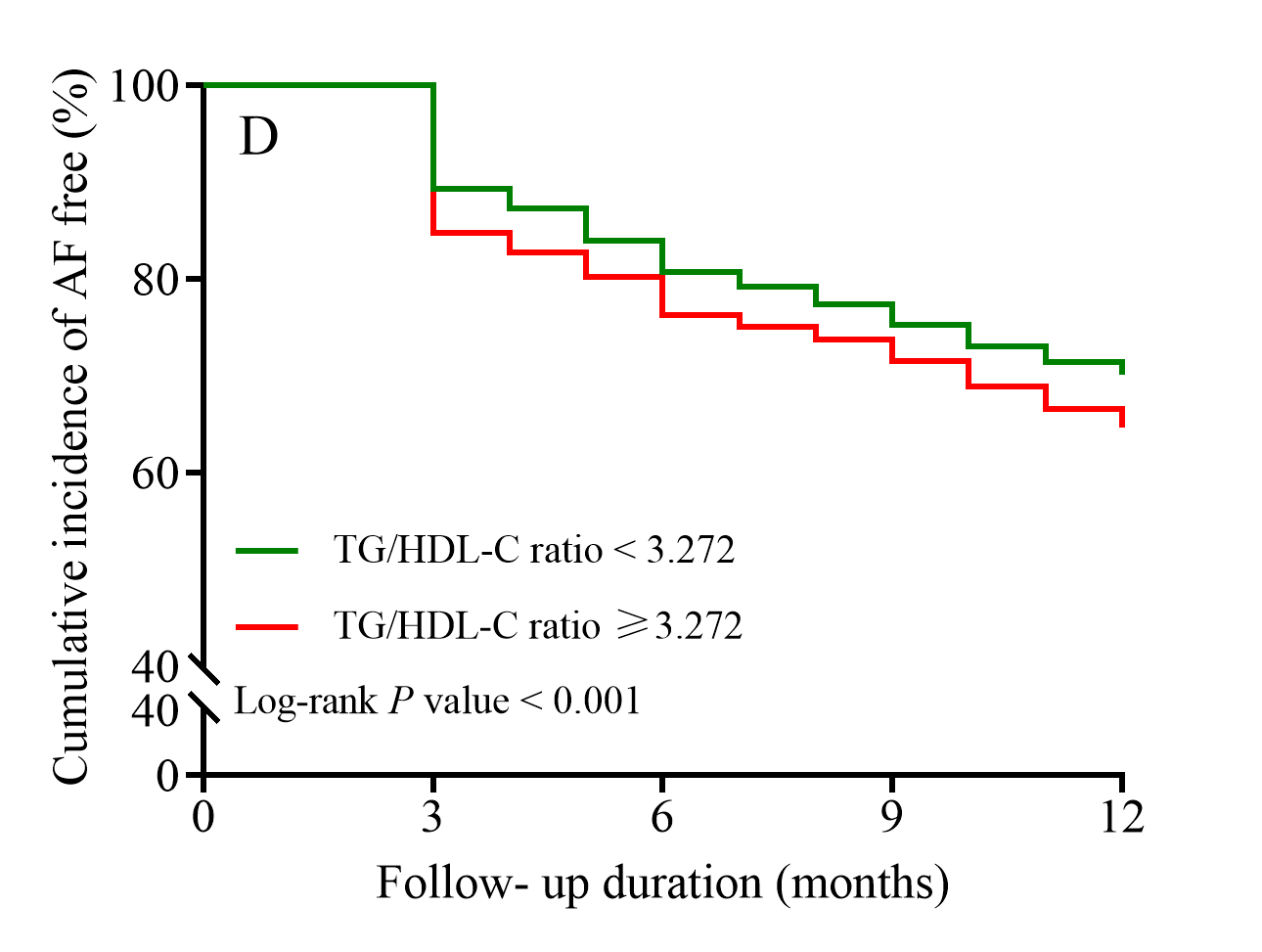

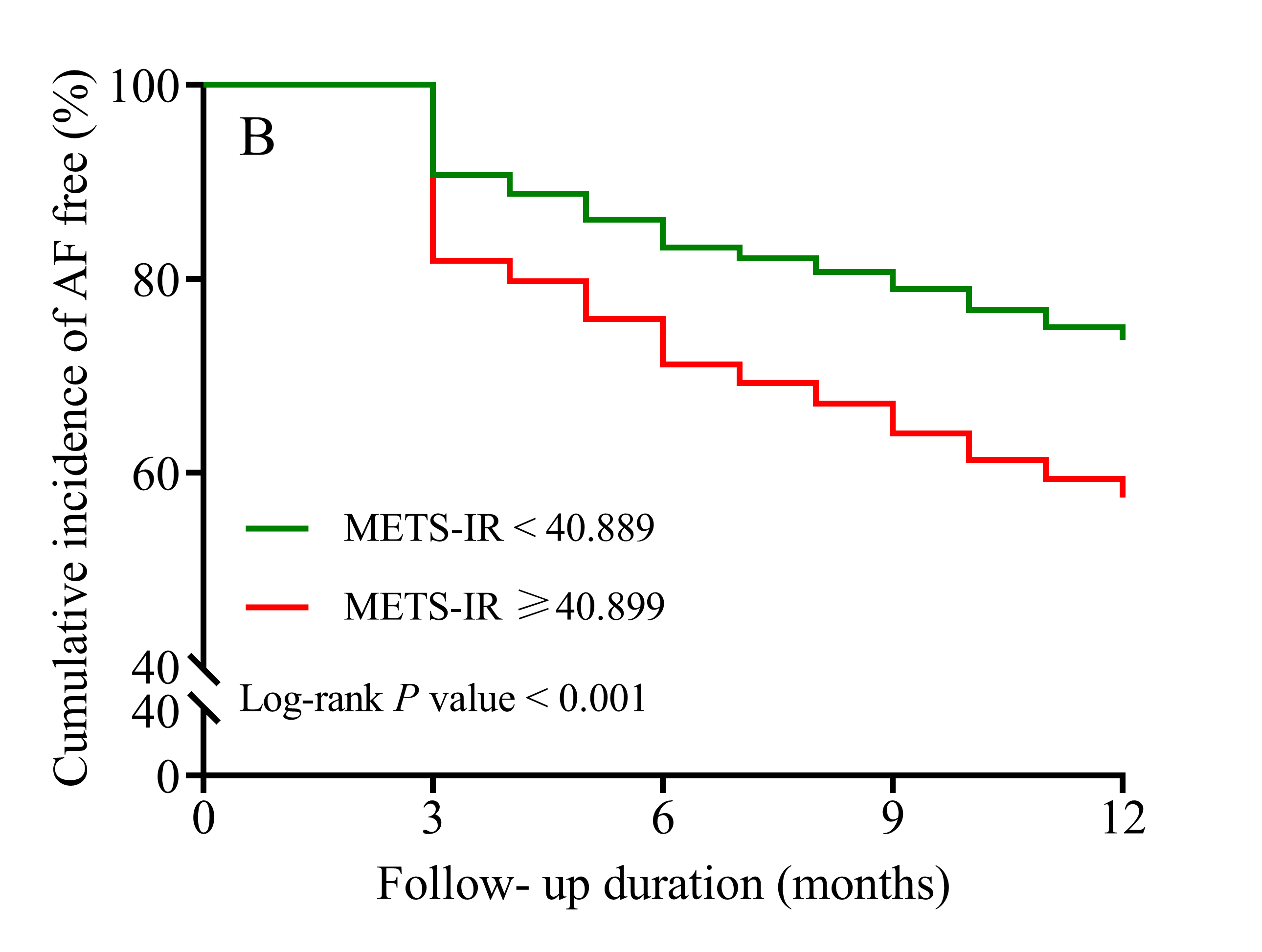

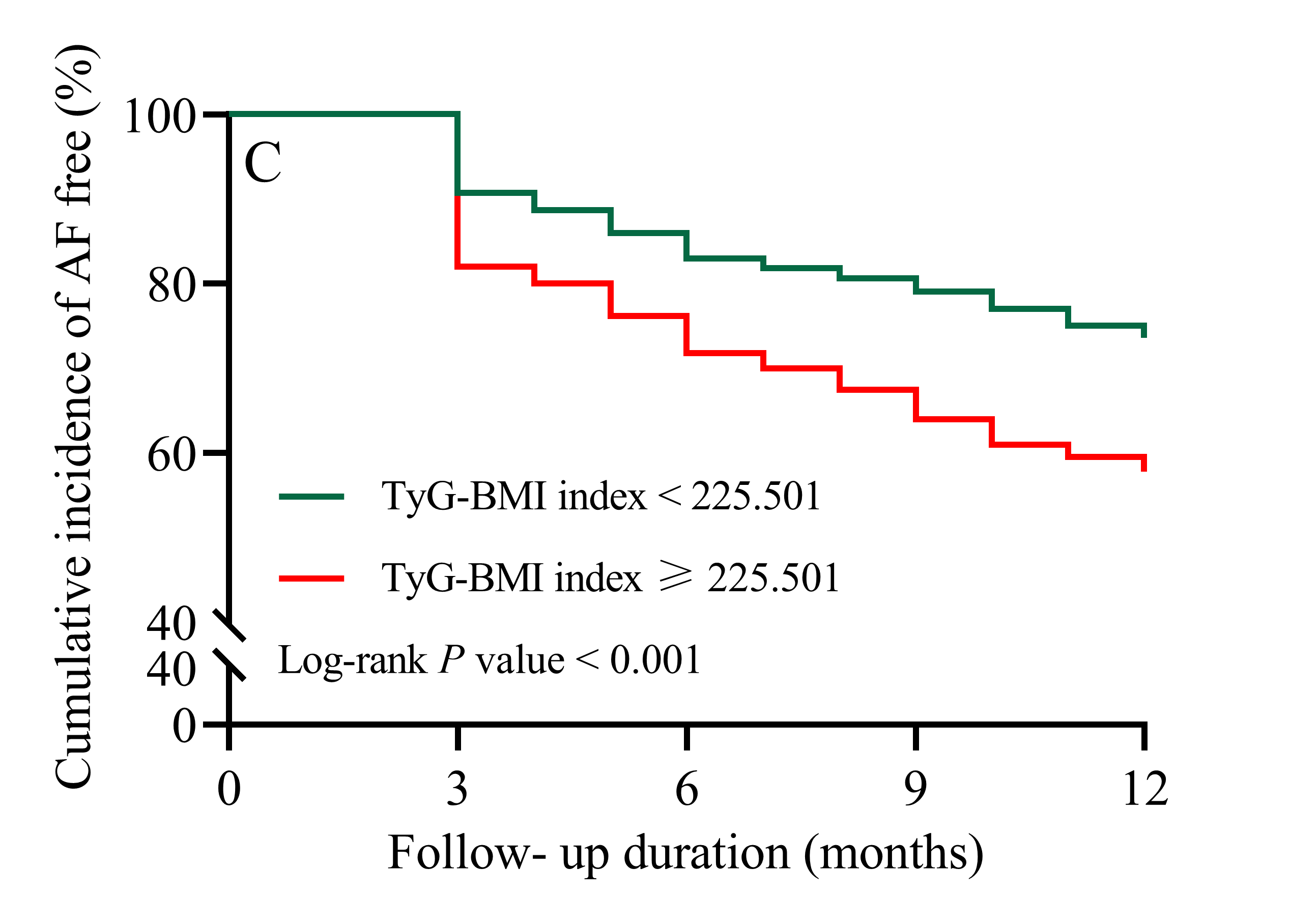

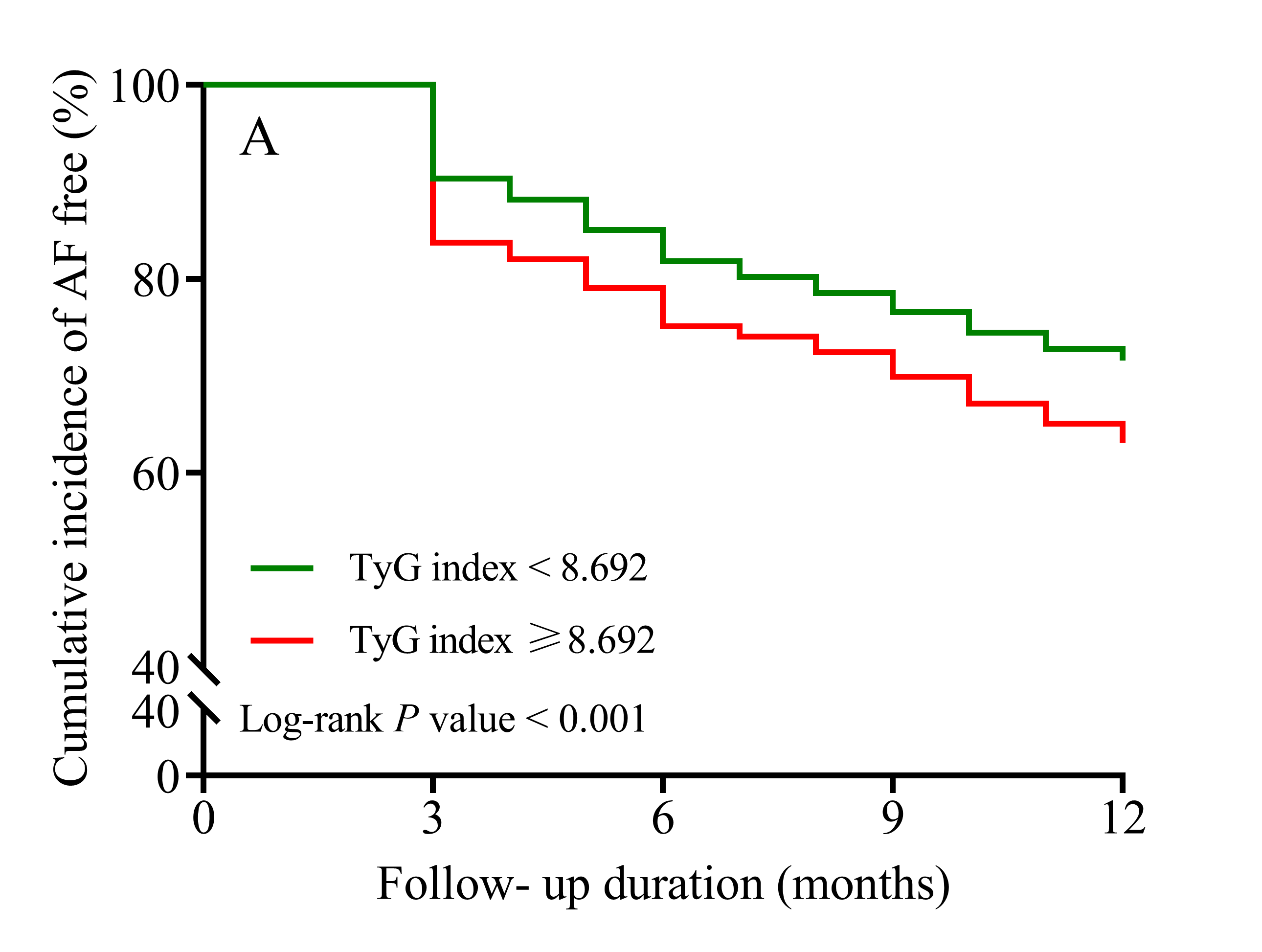


**Figure. S3** Kapla-Meier estimated event rates of AF recurrence following ablation according to cut-off values of ROC curves for insulin resistance indexes. METS-IR, metabolic score for insulin resistance; ROC, receiver operating curve; TyG, triglyceride and glucose; TyG-BMI, triglyceride glucose-body mass index; TG/HDL-C, triglyceride to high-density lipoprotein cholesterol


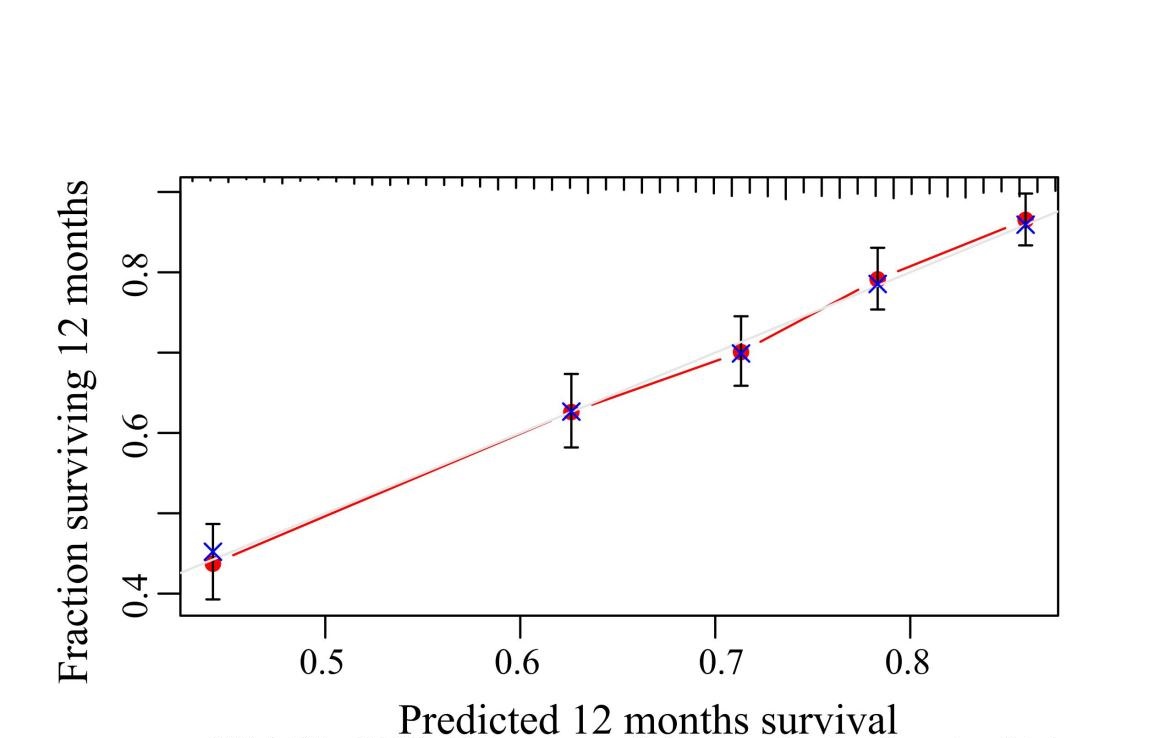


**Fig. S4** The calibration plots for the adjusted model predicting AF recurrence. The x-axis represents the predicted AF recurrence risk. The y-axis represents the actual AF recurrence rate. The gray line indicates a perfect prediction by an ideal model. The red solid line indicates the performance of the predicting model, of which a closer fit to the gray line suggests better prediction. The adjusted model refers to the established basic risk model. Established basic risk model included age, sex, body mass index, current drinking, diabetes mellitus, hyperlipidemia, coronary heart disease, duration of AF (≥24 months), AF type, fasting blood glucose, HbA1c, left atrial diameter, platelets, CHA_2_DS_2_-VASc score. AF, atrial fibrillation;


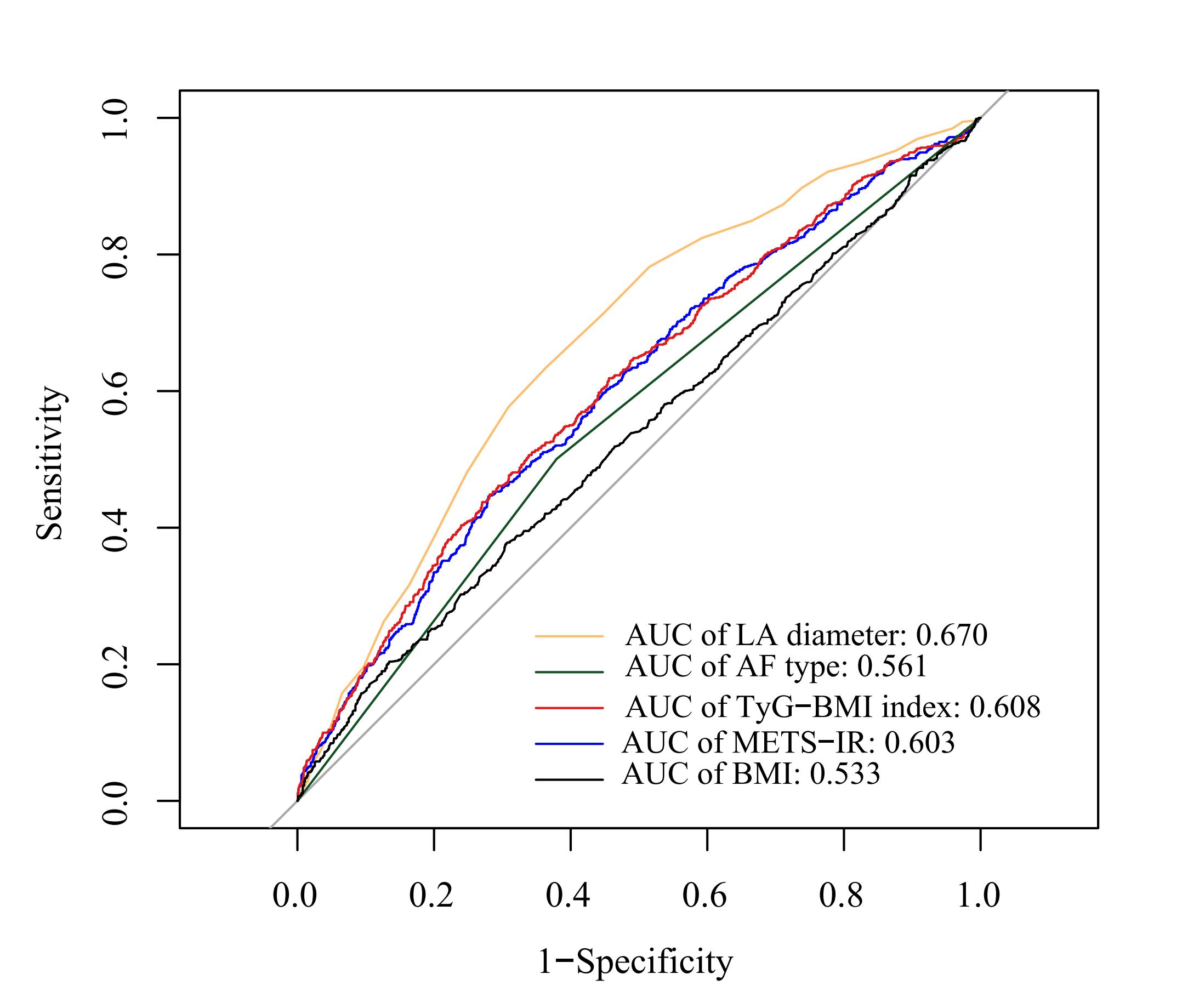


**Figure. S5** Receiver operating curve for risk factors in the detection of AF recurrence following ablation. AUC, area under curve; BMI, body mass index; METS-IR, metabolic score for insulin resistance; LA, left atrial; TyG-BMI, triglyceride glucose-body mass index

**
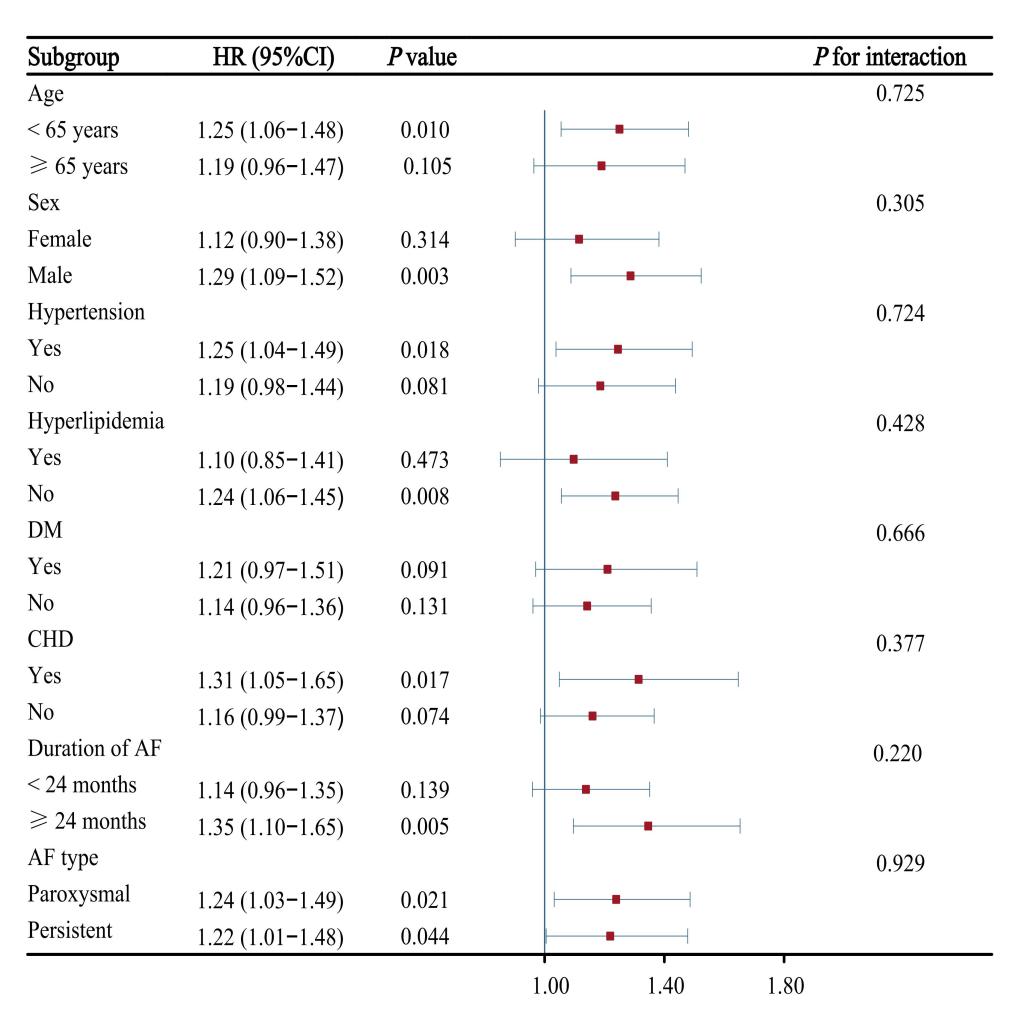
**

**Fig. S6** Association between TyG index (per 1 unit increase) and AF recurrence following ablation in different subgroups. AF, atrial fibrillation; CHD, coronary heart disease; DM, diabetes mellitus; TyG, triglyceride and glucose


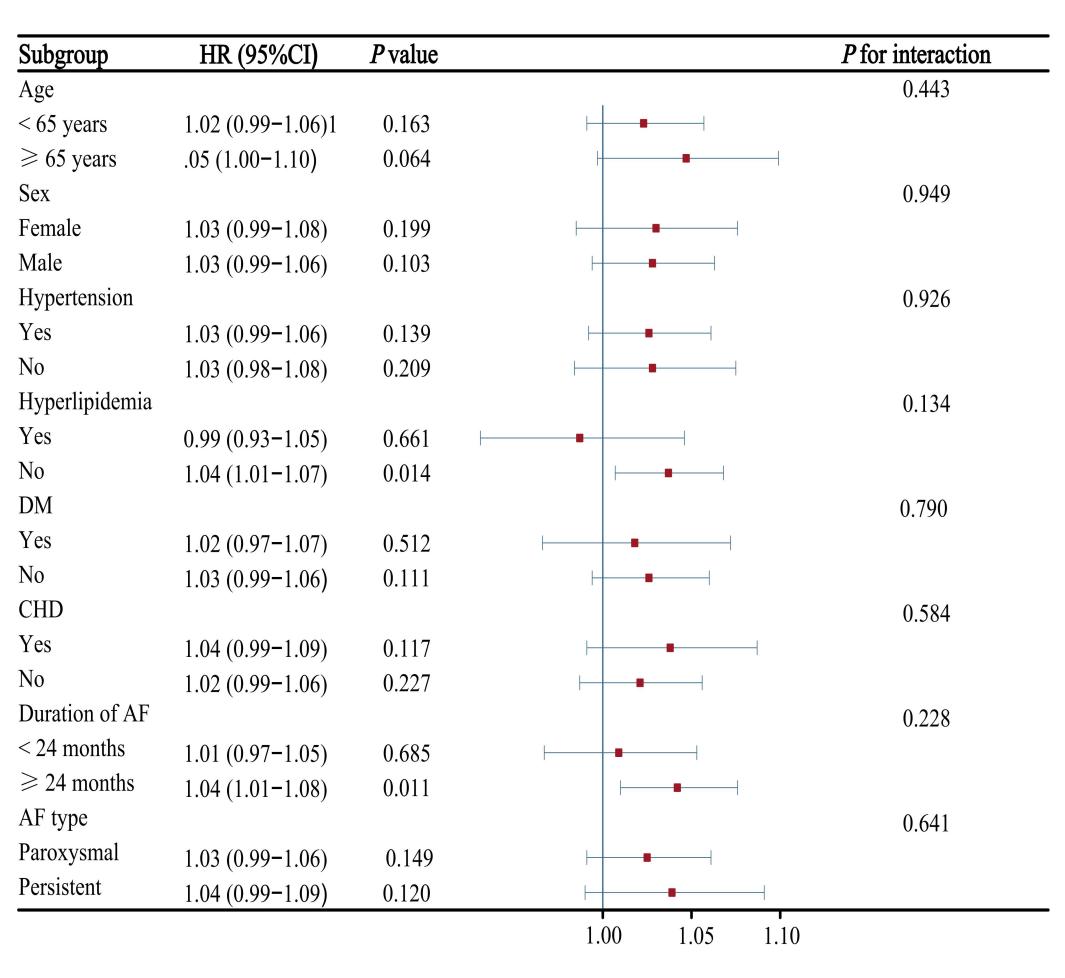


**Fig. S7** Association between TG/HDL-C ratio (per 1 unit increase) and AF recurrence following ablation in different subgroups. AF, atrial fibrillation; CHD, coronary heart disease; DM, diabetes mellitus; TG/HDL-C, triglyceride to high-density lipoprotein cholesterol


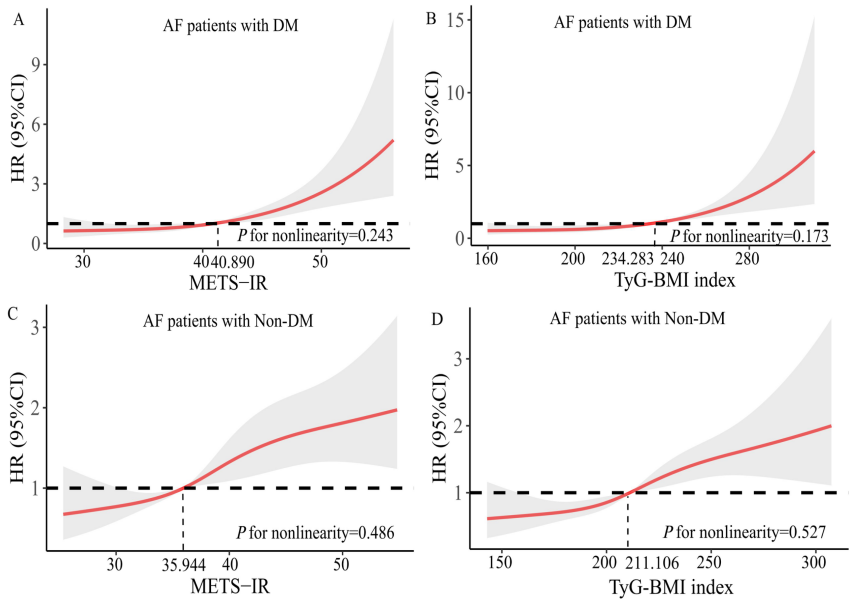


**Fig. S8** Restricted cubic spline curves for AF recurrence by METS-IR and TyG-BMI index after covariate adjustment in DM and non-DM patients. The risk factors adjusted for in this analysis included age, sex, current drinking, hyperlipidemia, coronary heart disease, duration of AF (≥ 24 months), AF type, HbA1c, left atrial diameter, platelet, CHA_2_DS_2_-VASc. AF, atrial fibrillation; CI, confidence interval; HR, hazard ratio; METS-IR, metabolic score for insulin resistance; TyG-BMI, triglyceride glucose-body mass index


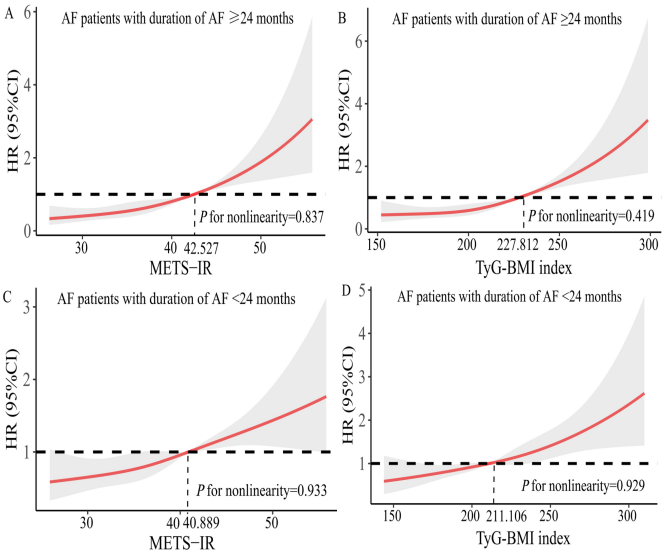


**Fig. S9** Restricted cubic spline curves for AF recurrence by METS-IR ans TyG-BMI index after covariates adjustment in duration of AF ≥ 24 and < 24 months patients. The risk factors adjusted for in this analysis included age, sex, current drinking, hyperlipidemia, coronary heart disease, diabetes mellitus, AF type, HbA1c, left atrial diameter, platelet, CHA_2_DS_2_-VASc. AF, atrial fibrillation; CI, confidence interval; HR, hazard ratio; METS-IR, metabolic score for insulin resistance; TyG-BMI, triglyceride glucose-body mass index

**Table S1** Association between non-insulin-based IR indexes and AF recurrence after ablation stratified by the statins medication at admission.

|  | **HR (95%CI)** | ***P* value** | ***P* for interaction** |
| --- | --- | --- | --- |
| TyG index (per 1 unit increase) | | | 0.239 |
| Statins | 1.09 (0.87-1.37) | 0.456 |  |
| Non-statins | 1.29 (1.10-1.52) | 0.002 |  |
| METS-IR (per 1 unit increase) | | | 0.974 |
| Statins | 1.04 (1.03-1.06) | <0.001 |  |
| Non-statins | 1.04 (1.03-1.06) | <0.001 |  |
| TyG-BMI index (per 10 unit increase) | | | 0.998 |
| Statins | 1.10 (1.06-1.14) | <0.001 |  |
| Non-statins | 1.10 (1.07-1.12) | <0.001 |  |
| TG/HDL-C ratio (per 1 unit increase) | | | 0.231 |
| Statins | 1.00 (0.94-1.06) | 0.893 |  |
| Non-statins | 1.04 (1.01-1.07) | 0.014 |  |

CI, confidence interval; HR, hazard ratio; IR, insulin resistance; METS-IR, metabolic score for insulin resistance; TyG, triglyceride and glucose; TyG-BMI, triglyceride glucose-body mass index; TG/HDL-C, triglyceride to high-density lipoprotein cholesterol

**Table S2** Association between METS-IR or TyG-BMI index with DM or non-DM and AF recurrence after ablation.

| **Variables** | **HR (95%CI)** | ***P* value** |  |
| --- | --- | --- | --- |
| METS-IR <40.899 without DM | Reference |  |  |
| METS-IR <40.899 with DM | 0.99 (0.75-1.32) | 0.962 |  |
| METS-IR ≥40.899 without DM | 1.42 (1.17-1.73) | <0.001 |  |
| METS-IR ≥40.899 with DM | 2.06 (1.57-2.70) | <0.001 |  |
| TyG-BMI index <225.501 without DM | Reference |  |  |
| TyG-BMI index <225.501 with DM | 0.99 (0.74-1.31) | 0.922 |  |
| TyG-BMI index ≥225.501 without DM | 1.37 (1.13-1.66) | 0.001 |  |
| TyG-BMI index ≥225.501 with DM | 1.94 (1.48-2.53) | <0.001 |  |

DM, diabetes mellitus; HR, hazard ratio; CI, confidence interval; METS-IR, metabolic score for insulin resistance; TyG-BMI, triglyceride glucose-body mass index

**Table S3** Added predictive ability and reclassification statistics of METS-IR and TyG-BMI index in DM and non-DM patients.

|  | **C-statistic**  **(95% CI)** | ***P* value** | **IDI (95% CI)** | ***P* value** | **Continuous**  **NRI (95% CI)** | ***P* value** |
| --- | --- | --- | --- | --- | --- | --- |
| AF patients with DM | | | | | | |
| Baseline risk model | 0.680 (0.637-0.723) | Reference | Reference |  | Reference |  |
| +METS-IR | 0.709 (0.667-0.752) | 0.042 | 0.061 (0.029-0.095) | 0.002 | 0.213 (0.099-0.292) | 0.002 |
| +TyG-BMI index | 0.711 (0668-0.753) | 0.041 | 0.071 (0.036-0.111) | <0.001 | 0.242 (0.156-0.312) | <0.001 |
| AF patients with non-DM | | | | | | |
| Baseline risk model | 0.695 (0.666-0.723) | Reference | Reference |  | Reference |  |
| +METS-IR | 0.702 (0.674-0.730) | 0.120 | 0.004 (-0.007-0.015) | 0.380 | 0.053 (-0.037-0.120) | 0.202 |
| +TyG-BMI index | 0.703 (0.675-0.731) | 0.111 | 0.006 (-0.005-0.018) | 0.264 | 0.094 (-0.003-0.147) | 0.060 |

AF, atrial fibrillation; CI, confidence interval; DM, diabetes mellitus; IDI, integrated discrimination improvement; METS-IR, metabolic score for insulin resistance; NRI, net reclassification improvement; TyG-BMI, triglyceride glucose-body mass index

Baseline risk model: age, sex, body mass index, current drinking, hyperlipidemia, coronary heart disease, duration of AF (≥ 24 months), AF type, fasting blood glucose, HbA1c, left atrial diameter, platelet, CHA_2_DS_2_-VASc score

**Table S4**Association between METS-IR or TyG-BMI index with duration of AF ≥ 24

or < 24months and AF recurrence after ablation.

| **Variables** | **HR (95%CI)** | ***P* value** |
| --- | --- | --- |
| METS-IR <40.899 with D1 | Reference |  |
| METS-IR <40.899 with D2 | 1.59 (1.29-1.96) | <0.001 |
| METS-IR ≥40.899 with D1 | 2.02 (1.59-2.56) | <0.001 |
| METS-IR ≥40.899 with D2 | 2.18 (1.74-2.74) | <0.001 |
| TyG-BMI index <225.501 with D1 | Reference |  |
| TyG-BMI index <225.501 with D2 | 1.56 (1.27-1.93) | <0.001 |
| TyG-BMI index ≥225.501 with D1 | 1.88 (1.49-2.38) | <0.001 |
| TyG-BMI index ≥225.501 with D2 | 2.10 (1.68-2.63) | <0.001 |

HR, hazard ratio; CI, confidence interval; METS-IR, metabolic score for insulin resistance; TyG-BMI, triglyceride glucose-body mass index. D1, duration of AF <24 months; D2, duration of AF ≥24 months

**Table S5** Added predictive ability and reclassification statistics of METS-IR and TyG-BMI index in duration of AF ≥ 24 and <24 months patients.

|  | **C-statistic**  **(95% CI)** | ***P* value** | **IDI (95% CI)** | ***P* value** | **Continuous**  **NRI (95% CI)** | ***P* value** |
| --- | --- | --- | --- | --- | --- | --- |
| AF patients with duration of AF ≥ 24 months | | | | | | |
| Baseline risk model | 0.699 (0.664-0.733) | Reference | Reference |  | Reference |  |
| +METS-IR | 0.723 (0.689-0.757) | 0.024 | 0.048 (0.023-0.072) | 0.002 | 0.191 (0.108-0.253) | 0.002 |
| +TyG-BMI index | 0.722 (0687-0.756) | 0.038 | 0.053 (0.028-0.081) | <0.001 | 0.212 (0.122-0.274) | <0.001 |
| AF patients with duration of AF < 24 months | | | | | | |
| Baseline risk model | 0.681 (0.649-0.714) | Reference | Reference |  | Reference |  |
| +METS-IR | 0.684 (0.651-0.717) | 0.525 | 0.004 (-0.010-0.018) | 0.669 | 0.068 (-0.049-0.134) | 0.244 |
| +TyG-BMI index | 0.686 (0.653-0.719) | 0.392 | 0.007 (-0.007-0.023) | 0.300 | 0.094 (-0.006-0.161) | 0.068 |

AF,atrial fibrillation; CI, confidence interval; IDI, integrated discrimination improvement; METS-IR, metabolic score for insulin resistance; NRI, net reclassification improvement; TyG-BMI, triglyceride glucose-body mass index

Baseline risk model: age, sex, body mass index, current drinking, hyperlipidemia, coronary heart disease, diabetes mellitus, AF type, fasting blood glucose, HbA1c, left atrial diameter, platelet, CHA_2_DS_2_-VASc score
